# Air Cleaners and Respiratory Infections in Schools: A Modeling Study Based on Epidemiologic, Environmental, and Molecular Data

**DOI:** 10.1093/ofid/ofae169

**Published:** 2024-03-21

**Authors:** Nicolas Banholzer, Philipp Jent, Pascal Bittel, Kathrin Zürcher, Lavinia Furrer, Simon Bertschinger, Ernest Weingartner, Alban Ramette, Matthias Egger, Tina Hascher, Lukas Fenner

**Affiliations:** Institute of Social and Preventive Medicine, University of Bern, Bern, Switzerland; Multidisciplinary Center for Infectious Diseases, University of Bern, Bern, Switzerland; Multidisciplinary Center for Infectious Diseases, University of Bern, Bern, Switzerland; Department of Infectious Diseases, Inselspital, Bern University Hospital, University of Bern, Bern, Switzerland; Multidisciplinary Center for Infectious Diseases, University of Bern, Bern, Switzerland; Institute for Infectious Diseases, University of Bern, Bern, Switzerland; Institute of Social and Preventive Medicine, University of Bern, Bern, Switzerland; Multidisciplinary Center for Infectious Diseases, University of Bern, Bern, Switzerland; Institute for Infectious Diseases, University of Bern, Bern, Switzerland; Institute of Social and Preventive Medicine, University of Bern, Bern, Switzerland; Institute for Sensors and Electronics, University of Applied Sciences and Arts Northwestern Switzerland, Windisch, Switzerland; Multidisciplinary Center for Infectious Diseases, University of Bern, Bern, Switzerland; Institute for Infectious Diseases, University of Bern, Bern, Switzerland; Institute of Social and Preventive Medicine, University of Bern, Bern, Switzerland; Population Health Sciences, University of Bristol, Bristol, UK; Centre for Infectious Disease Epidemiology and Research, University of Cape Town, Cape Town, South Africa; Multidisciplinary Center for Infectious Diseases, University of Bern, Bern, Switzerland; Institute of Educational Science, University of Bern, Bern, Switzerland; Institute of Social and Preventive Medicine, University of Bern, Bern, Switzerland; Multidisciplinary Center for Infectious Diseases, University of Bern, Bern, Switzerland

**Keywords:** air cleaner, airborne transmission, molecular detection, respiratory viruses, schools

## Abstract

**Background:**

Using a multiple-measurement approach, we examined the real-world effectiveness of portable HEPA air filtration devices (air cleaners) in a school setting.

**Methods:**

We collected data over 7 weeks during winter 2022/2023 in 2 Swiss secondary school classes: environmental (CO_2_, particle concentrations), epidemiologic (absences related to respiratory infections), audio (coughing), and molecular (bioaerosol and saliva samples). Using a crossover design, we compared particle concentrations, coughing, and risk of infection with and without air cleaners.

**Results:**

All 38 students participated (age, 13*–*15 years). With air cleaners, mean particle concentration decreased by 77% (95% credible interval, 63%*−*86%). There were no differences in CO_2_ levels. Absences related to respiratory infections were 22 without air cleaners vs 13 with them. Bayesian modeling suggested a reduced risk of infection, with a posterior probability of 91% and a relative risk of 0.73 (95% credible interval, 0.44*–*1.18). Coughing also tended to be less frequent (posterior probability, 93%), indicating that fewer symptomatic students were in class. Molecular analysis detected mainly non–SARS-CoV-2 viruses in saliva (50/448 positive) but not in bioaerosols (2/105) or on the HEPA filters of the air cleaners (4/160). The molecular detection rate in saliva was similar with and without air cleaners. Spatiotemporal analysis of positive saliva samples identified several likely transmissions.

**Conclusions:**

Air cleaners improved air quality and showed potential benefits in reducing respiratory infections. Airborne detection of non–SARS-CoV-2 viruses was rare, suggesting that these viruses may be more difficult to detect in the air. Future studies should examine the importance of close contact and long-range transmission and the cost-effectiveness of using air cleaners.

## Teaser

Using a multiple-measurement approach in a school setting, we found that air cleaners improved air quality and showed a potential benefit in reducing non–SARS-CoV-2 respiratory infections.

Transmission of respiratory infections such as SARS-CoV-2 and influenza are difficult to mitigate and control. Person-to-person transmission occurs primarily through the release of respiratory particles containing the viruses. In the wake of the COVID-19 pandemic, infection control has focused on reassessing the role of small respiratory particles called *aerosols*, which have been found to carry the majority of viruses during respiratory activities [1]. Unlike larger respiratory droplets, which tend to settle quickly, aerosols can remain suspended in the air for several hours and travel long distances [2]. The relative importance of aerosols, droplets, and fomites to overall transmission is a matter of ongoing debate.

Improved ventilation systems are critical for a healthy indoor environment and can reduce the risk of respiratory transmission [3, 4], especially in schools where students spend most of their time indoors during the week. Portable HEPA air filtration devices (air cleaners) may be another cost-effective alternative to upgrading ventilation systems, but their impact on respiratory viral transmission is less clear. A population-level study reported a lower incidence of SARS-CoV-2 in US elementary schools using different ventilation strategies, including air filtration devices [5]. While several studies showed that air cleaners reduce particle concentrations [6–8], an association with viral RNA load in airborne samples could not be found [9, 10], although recent studies found that air cleaners effectively removed SARS-CoV-2 bioaerosols in hospitals and other indoor settings [11–13]. Simulation studies have further demonstrated the efficacy of air cleaners in reducing the risk of indoor transmission of SARS-CoV-2 [14] and other respiratory viruses [15]. However, most simulation studies are based on the assumption that detection of RNA equals transmissible virus, despite recent data revealing a relevant loss of viral infectivity in respiratory particles over time [16]. To date, it remains unclear whether reducing particle concentrations and removing bioaerosols will reduce indoor transmission of respiratory infections.

We used a multiple-measurement approach to study transmission of respiratory viruses under nonpandemic conditions and the effect of air cleaners in a school setting, using a crossover study design in the winter of 2022 to 2023. We collected the following data during a 7-week study from January to March 2023 in 2 Swiss secondary school classes: environmental (CO_2_, particle concentrations), epidemiologic (absences likely related to respiratory infections), audio (coughing), and molecular (detection of viruses in bioaerosol and saliva samples). We determined changes in particle concentrations, absences related to respiratory infections, coughing, and the rate of positive saliva samples.

## METHODS

This study is reported per the STROBE guideline (Strengthening the Reporting of Observational Studies in Epidemiology; see checklist in Supplementary material).

### Study Setting and Design

**Figure 1.**
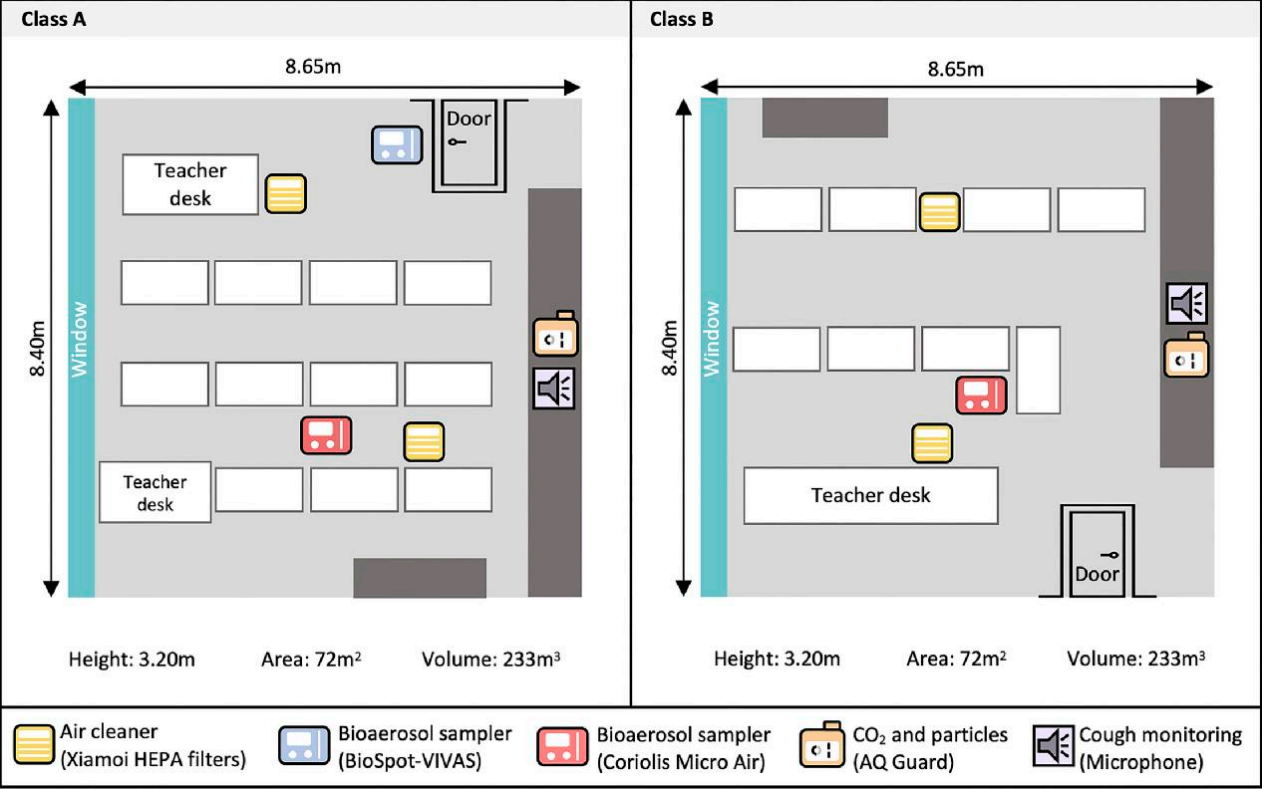
Study setting: schematic setup of the classrooms. One air cleaner was placed in the front of the classrooms and one in the back. All devices were placed at the head level of the students when they were seated. Both classrooms lacked an active HVAC system (heating, ventilation, air conditioning), but they were ventilated naturally by opening windows at the discretion of the teachers.

We collected data in 2 classrooms of a secondary school (age of students, 14–17 years) in the canton of Solothurn, Switzerland, for 7 weeks from 16 January to 11 March 2023. Figure 1 shows the schematic study setup.

### Study Intervention

We used a crossover design to study the effectiveness of air cleaners (Table 1). Air cleaners refer to commercially available portable HEPA filtration devices (Xiaomi Mi Air Pro 70 m^2^). According to the manufacturer, these air cleaners run at clean air delivery rates of 2 *×* 600 m^3^/h. When testing the devices in an empty classroom with submicrometer-sized particles, we measured a lower effective clean air delivery rate of 2 *×* 420 m^3^/h (Supplementary Text A).

**Table 1.** Crossover Study Design.

	Week 1	Week 2	Week 3	Vacation	Week 4	Week 5	Week 6	Week 7
Class	16–21 Jan	23–28 Jan	30 Jan–3 Feb	6–11 Feb	13–18 Feb	20–25 Feb	27 Feb–3 Mar	6–11 Mar
A	PAC	PAC ^a^	None	…	None	None	PAC	PAC ^a^
B	None	None	PAC ^a^	…	PAC	PAC ^a^	None	None

Description of when PACs were installed in the rooms of classes A and B during a 7-week study period from 16 January to 11 March 2023, excluding a week of vacation from 6 to 11 February.

Abbreviation: PAC, portable air cleaner.

^a^Swabs from the HEPA filters were taken after each intervention phase and before the vacation.

### Data Collection

An overview of the collected data is provided in Supplementary Text B and Supplementary Table 1.

#### Environmental Data

An air quality device (AQ Guard; Palas GmbH) continuously measured indoor CO_2_ levels, aerosol number (particle diameter, 175 nm–20 µm), and particle mass concentrations (PM_1_, PM_2.5_, PM_4_, PM_10_) by minute [8].

#### Epidemiologic Data

At study start, we collected aggregated data on age, sex, COVID-19 vaccination, and recovery status in the participating classes. We also collected daily data on each absent student. For absences due to illness, we recorded symptoms and date of symptom onset. We defined a case of respiratory infection as an absence in which the student reported an illness with at least 1 respiratory symptom (Supplementary Text C). All absences are listed in Supplementary Table 2.

#### Audio Recordings and Cough Detection

We installed portable audio recorders (ZOOM H6) to record sounds continuously. We determined the number of coughs per minute using an artificial intelligence algorithm [17]. A recent study showed a significant correlation between coughing and airborne viral detection [10]. Also, coughing can serve as a proxy for the prevalence of symptomatic infection among students in the classroom.

#### Molecular Data Analyses

Both classes participated in repetitive biweekly saliva testing (Tuesdays and Thursdays). Samples were transported to the laboratory and stored at *−*80 °C until further processing [18]. All positive samples are listed in Supplementary Table 3 and Supplementary Text D. Furthermore, we collected airborne respiratory viruses in both classrooms with a cyclonic bioaerosol sampling device (Coriolis Micro Air; Bertin Instruments), running at 200 L/min and collecting into 15 mL of phosphate-buffered saline. The Coriolis Micro Air ran shortly before and during break times (approximately 60 min/d) to minimize noise. In one class, we also sampled with the BioSpot-VIVAS condensation particle growth collection device (Aerosol Devices Inc) [19], which operated throughout lessons. The removable parts were regularly autoclaved. At the end of the day, samples were transported to the Institute of Infectious Diseases and stored at *−*80 °C. Finally, we collected swabs from the HEPA filters of the air cleaners after each intervention phase (Table 1). The HEPA filters were removed and divided into 20 fields. One sterile phosphate-buffered saline–moistened swab per field was taken for a total of 20 swabs per filter.

Prior to the real-time polymerase chain reaction analysis, daily bioaerosol samples were combined for each sampling device and filtered through Amicon Ultra-15 Centrifugal Filters with Ultracel filters with molecular weight cutoffs of 10 000 Da (UFC9010; MilliporeSigma) to a volume of 1 mL. Saliva samples were analyzed directly without prior filtration. The Allplex RV Master Assay (Seegene) detects a panel of 19 major respiratory viruses and viral subtypes, including SARS-CoV-2, influenza, respiratory syncytial virus, metapneumovirus, adenovirus, rhinovirus, and parainfluenza.

We also performed molecular genotyping for positive saliva, bioaerosol, and air filter samples of adenovirus and influenza [20].

### Statistical Analyses and Modeling

All statistical analyses were described in a statistical analysis plan [21]. Bioaerosol samples and viral load concentrations could not be analyzed because there were too few positive samples. Saliva samples were used only for surveillance of respiratory viruses. A comparison of the saliva positivity rate between study conditions was not performed because saliva sampling was anonymous and the same student could thus be detected multiple times. Further minor deviations from the statistical analysis plan are documented in Supplementary Texts E to G, including detailed descriptions of the models.

#### Particle Concentrations

We compared daily mean particle concentrations between study conditions (Supplementary Figure 1). We estimated the reduction in particle concentrations with air cleaners using bayesian log-linear regression models, adjusting for observed confounders (Supplementary Text E).

#### Risk of Infection

We estimated the relative risk of infection with air cleaners using a bayesian latent variable regression model (Supplementary Text F). The number of new respiratory cases *C* (observed absences related to respiratory infections by date of symptom onset) on day *t* in class *j* are modeled with a negative binomial distribution. The expected number of new cases is the weighted sum of the number of new infections *I_js_* (latent variable) in the previous days *s < t*, with the weights corresponding to the probability distribution of the incubation period (Supplementary Figure 2). The incubation period was approximated with a weighted mixture of the incubation periods of the viruses detected in saliva in the same week as the absences. The number of new infections is related to the presence of portable air cleaners as follows:


(1) $$\log I_{\;js}=\log F_{\;js}-\log N_{\;js}+\beta_{0}+\beta_{1}\cdot {\mathrm{PAC}}_{\;js},$$

where *F_js_* is the number of infections in the previous week (a proxy for the number of infectious students), *N_js_* is the cumulative number of infections (a proxy for the number of susceptible students), β_0_ is the infection rate without air cleaners, and β_1_ is the effect of air cleaners. Furthermore, the effect of air cleaners is adjusted for class-specific effects, the number of students in class, the daily air change rate, and the weekly positivity rate for COVID-19 and the consultations for influenza-like illnesses in the canton. A higher prevalence of respiratory illnesses in the community was a proxy for possible higher exposure to respiratory infections outside the school.

#### Coughing

We estimated the reduction in the daily number of coughs with air cleaners in a bayesian negative binomial regression model, using time in class as the model offset and adjusting for observed confounders (Supplementary Text G). In addition, we estimated the association between the number of coughs and the virus-specific number of positive saliva samples, using only the days when saliva samples were collected (Tuesdays and Thursdays, for a total of 27 days).

#### Software

All analyses were performed in R (version 4.2.0) and model parameters estimated in Stan (version 2.21.0) [22, 23]. For each outcome, we report the posterior probability of a reduction with air cleaners. The estimated reduction is reported with the posterior mean and 95% credible interval (95% CrI).

### Patient Consent Statement

The Ethics Committee of the canton of Bern, Switzerland, approved the study (reference 2021-02377). For the saliva samples, we included all students who were willing to participate and obtained written informed consent from their caregivers.

## RESULTS

The study population consisted of 38 students (age, 13*–*15 years; 19/19 female/male; Table 2). Seven students had been vaccinated against or had recovered from a SARS-CoV-2 infection within the last 4 months. During the 7-week study period (1330 student-days), students were absent from school for 220 days (18%), of which 129 (59%) were due to illness.

**Table 2.** Study Population and Person-Days of Absences

	Class A	Class B	Total
Students	20 (53)	18 (47)	38 (100)
Gender			
Female	11 (55)	8 (44)	19 (50)
Male	9 (45)	10 (56)	19 (50)
Immunity status			
Recently vaccinated or recovered	7 (39)	0 (0)	7 (18)
Not recently vaccinated or recovered	11 (61)	20 (100)	31 (82)
Absent person-days	110 (50)	110 (50)	220 (100)
Sickness	52 (47)	77 (70)	129 (59)
Other	58 (53)	33 (30)	91 (41)

Data are presented as No. (%).

### Air Quality

**Figure 2.**
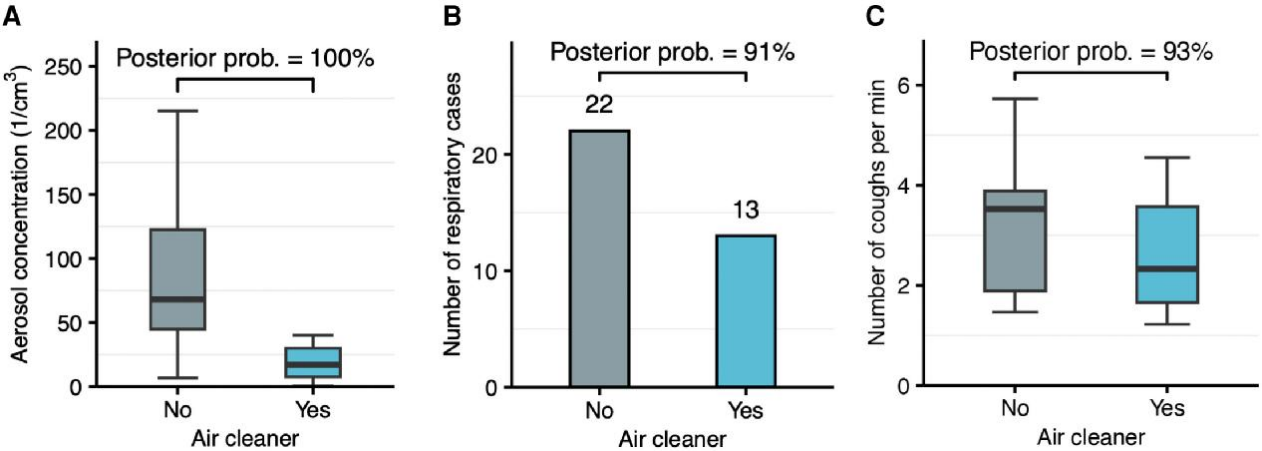
Comparison of outcomes with and without air cleaners. The top of each plot shows the posterior probability for a reduction with air cleaners based on the bayesian model. *A*, Daily average aerosol number concentrations. *B*, Number of respiratory cases. *C*, Daily average number of detected coughs per minute. Line, median; box, interquartile range (IQR); error bars, lower and upper value no further than 1.5 times IQR.

The mean aerosol number concentration without air cleaners was 95 1/cm^3^ (SD, 81) vs 27 1/cm^3^ (SD, 34 1/cm^3^) with air cleaners (Figure 2*A*). The bayesian regression model suggested a clear reduction in aerosol concentration with air cleaners, with a posterior probability of 100%. The model-estimated decrease was 76% (95% CrI, 63%–86%), which was greater for larger particles (PM_10_) than for smaller ones (PM_1*–*4_; Supplementary Figure 3, Supplementary Table 4). Daily mean CO_2_ levels were comparable between study conditions: 1636 ppm (SD, 341) without air cleaners vs 1769 ppm (SD, 391) with them. There was little change in other environmental variables (Supplementary Figure 4).

### Risk of Infection

Absences related to respiratory infections included 22 cases without air cleaners vs 13 cases with them (Figure 2*B*). The bayesian latent variable hierarchical regression model suggested that air cleaners reduced the risk of infection, with a posterior probability of 91%. The adjusted relative risk of infection with air cleaners was 0.73 (95% CrI, 0.44–1.18). The estimated number of respiratory infections in school would have been 19 (95% CrI, 9–37) if air cleaners had been installed throughout the study period, as compared with 36 (95% CrI, 12–92) if air cleaners had not been installed. Detailed estimation results are provided in the supplementary information (Supplementary Text J, Supplementary Figure 5, Supplementary Table 5).

### Coughing

On average, we detected 3.1 coughs/min (SD, 1.2) without air cleaners vs 2.6 coughs/min (SD 1.1) with them (Figure 2*C*). The bayesian model suggested that coughing was less frequent with air cleaners, with a posterior probability of 93%. The adjusted relative risk of coughing with air cleaners was 0.93 (95% CrI, 0.85–1.02). Coughing was associated with virus-specific transmission (Supplementary Figure 6).

### Saliva Samples

We analyzed 448 saliva samples. In terms of number of virus-positive samples, we detected 15 influenza B, 15 rhinovirus, 14 adenovirus, 3 SARS-CoV-2, 2 metapneumovirus, and 1 parainfluenza. The distribution of positive saliva samples varied between classes. For example, all but 1 sample was positive for adenovirus in class A during the first 3 study weeks, while most positive influenza B samples were found in class B. To illustrate possible transmission chains within classes, we linked positive saliva samples of the same virus that were <1 week apart, identifying 10 possible transmission chains (Supplementary Figure 7). Molecular genotyping to verify the proposed transmission network was unsuccessful because we could not amplify and sequence any gene targets. We also analyzed 105 bioaerosol samples and detected viral RNA in 2: 1 rhinovirus in class A and 1 adenovirus in class B. Similarly, we detected 1 influenza B, 1 rhinovirus, 1 adenovirus, and 1 SARS-CoV-2 in the 20 swabs taken from each filter of an air cleaner after each intervention phase (160 swabs total).

## DISCUSSION

We used a multiple-measurement approach within a crossover study design to estimate the risk of respiratory virus infection in a Swiss school and to assess the effectiveness of air cleaners. We found a range of respiratory viruses in saliva samples, mainly adenovirus, influenza B, and rhinovirus, but very little viral RNA was detected in bioaerosol samples and on the filters of the air cleaners. PM concentrations decreased significantly with air cleaners, and bayesian modeling based on epidemiologic data was compatible with a small reduction in the relative risk of infection with air cleaners. Coughing was reduced, indicating a lower prevalence of symptomatic infections with air cleaners.

Notably, we detected only 3 positive SARS-CoV-2 saliva samples. In a previous similar study in the same setting, we estimated the effectiveness of mask wearing and air cleaners during the SARS-CoV-2 Omicron wave in the winter of 2021 to 2022 and detected almost exclusively SARS-CoV-2 in the students’ saliva [8]. A similar shift in the pattern of respiratory viruses has been observed in other studies [24, 25]. We found a reduction in the risk of SARS-CoV-2 infection for mask wearing but not for air cleaners, possibly because the air cleaners were introduced at the end of the study, when most students were already infected with SARS-CoV-2.

It is well documented that air cleaners improve indoor air quality [6–8]. There are several reasons why the effect of air cleaners is probably smaller than universal mask wearing, which has been shown to be a very effective infection control measure [5, 8, 26, 27]. Unlike masks, air cleaners cannot prevent transmission outside the classroom or transmission due to close-range, high-particle density. Prolonged and close contact may be necessary for transmission of some respiratory viruses [26, 28] or make transmission more likely despite prior vaccination or infection [29]. Our results further suggest that air cleaners are more effective at removing larger particles (>5 µm), which also explains the difference between our measured clean air delivery rate and the manufacturer's reported rate (Supplementary Text A). However, many respiratory viruses are carried in smaller particles, which are more relevant for transmission (≤5 µm) [1]. Finally, classroom activity, airflow, and other unobserved confounding factors make it challenging to evaluate the effects of air cleaners on transmission in real-world settings.

The beneficial effects of air cleaners on indoor air quality and transmission come at a reasonable cost. The portable air cleaners used in our study cost approximately US $250 per unit. Their operating cost-effectiveness in providing clean air could be even higher than that of a ventilation system when compared in parallel with the same air delivery ratings [30]. Therefore, air cleaners could be a cost-effective public health measure, particularly during pandemics or epidemics when there is greater exposure to respiratory infections and greater concern of becoming infected [31]. However, their acceptance may be hindered by noise, space limitations, technical issues, and maintenance requirements [32]. Therefore, investments in professional building ventilation systems are still preferred in the long run [33].

We detected few respiratory viruses in bioaerosol samples (1 sample of adenovirus and 1 sample of rhinovirus) and on the filters of the air cleaners (4 positive samples). The low rate of positive bioaerosol samples may indicate that it is unlikely that airborne transmission occurred in classrooms. It may be possible that students had relatively little exposure to respiratory viruses at school and acquired their infections elsewhere. However, the distribution of positive saliva samples markedly differed between classes. Adenovirus spread in class A during the first 3 study weeks, with just 2 infections of influenza B over the study period. In contrast, influenza B spread throughout the study in class B, and adenovirus infections were detected only in the last week of the study. Furthermore, adenovirus infections tend to be mild [34] and less frequently associated with cough than influenza [35], consistent with the comparatively lower frequency of coughing in class B. Taken together, the class-specific spatiotemporal patterns indicate that transmission of respiratory infections may have occurred within the classrooms.

Our study has limitations. Aerosol measurements and molecular detection of viruses in bioaerosol samples document exposure but not transmission or its direction (person to air, air to person). Furthermore, the transmission route (aerosols, droplets, or fomites) cannot be determined by these measurements. The reasons for school absences were self-reported by students, and some absences may have been incorrectly attributed to respiratory infections. Unmeasured confounding, such as exposure to respiratory infections outside of school, also cannot be excluded. In addition, we could only approximate the incubation period for each epidemiologic case. Finally, although the study results likely apply to many settings in Switzerland and other European countries, they will not apply to settings in the global south.

In conclusion, a range of respiratory viruses, but rarely SARS-CoV-2, was detected in students under nonpandemic conditions when public health measures were lifted. Airborne detection was rare, suggesting that respiratory viruses other than SARS-CoV-2 may be more difficult to detect and that prolonged close contact may be required for transmission. The risk reduction of respiratory infections conferred by air cleaners may be modest at the individual level, but the benefit at the population level in terms of prevented illness and absences is likely to be important. Future studies should examine the cost-effectiveness of using air cleaners in congregate settings.

## Supplementary Data

Supplementary materials are available at *Open Forum Infectious Diseases* online. Consisting of data provided by the authors to benefit the reader, the posted materials are not copyedited and are the sole responsibility of the authors, so questions or comments should be addressed to the corresponding author.

## Supplementary Material

ofae169_Supplementary_Data

## Notes

***Acknowledgments.*** We thank the schools, teachers, and students participating in the study. We are grateful to the educational department of the canton of Solothurn for its support throughout the study. We also thank Ronald Dijkman for the influenza molecular genotyping and the biosecurity team (Julia Feldmann, Monika Gsell, Kathrina Summermatter) from the Institute for Infectious Diseases at the University of Bern for their assistance with the bioaerosol devices. Finally, we are indebted to the student assistants who helped with the data collection in the schools (Khadija Ahmed, Marie-Joséphine Brancato, Michelle Bürki, Santiago Martinez, Moric Toszeghi, Sylvain Wasmer).

***Author contributions.*** Conception and design: N. B., K. Z., L. Fenner, P. B., P. J., T. S. Epidemiological and environmental data collection: E. W., N. B., P. J., K. Z., T. H., L. Fenner. Laboratory data collection: P. B., L. Furrer. Additional data collection: T. H. Cough detection: S. B. Molecular genotyping: A. R., P. B., L. Furrer. Statistical analysis: N. B. Manuscript draft: N. B., M. E., L. Fenner. All authors reviewed and approved the final version of the manuscript.

***Data availability.*** Preprocessed data and code are available from https://osf.io/38j9g.

***Disclaimer***. The funder of the study had no role in study design, data collection, data analysis, data interpretation, or writing of the report.

***Financial support*.** This work was supported by the Multidisciplinary Center for Infectious Diseases, University of Bern, Switzerland. NB and L. Fenner are supported by the National Institute of Allergy and Infectious Diseases (NIAID) through cooperative agreement 5U01-AI069924-05. ME is supported by special project funding from the Swiss National Science Foundation (grant 32FP30-189498).
