# Supplementary material for: Air Cleaners and Respiratory Infections in Schools: A Modeling Study Based on Epidemiologic, Environmental, and Molecular Data
Source: Open Forum Infect Dis. 2024 Mar 21;11(4):ofae169. doi: 10.1093/ofid/ofae169 (PMC11045022; doi:10.1093/ofid/ofae169)
Supplement: ofae169_Supplementary_Data [file ofae169_supplementary_data.zip › revised-supplements.pdf]

Supplementary Material

Air cleaners and respiratory infections in schools: A multi-measurement observational study

Nicolas Banholzer[1‡,2], Philipp Jent[2,3], Pascal Bittel[2,4], Kathrin Zürcher[1], Lavinia Furrer[4], Simon Bertschinger[1], Ernest Weingartner[5], Alban Ramette[2,4], Matthias Egger[1,6,7], Tina Hascher[8], and Lukas Fenner[1*,2]

[1]Institute of Social and Preventive Medicine, University of Bern, Bern, Switzerland

[2]Multidisciplinary Center for Infectious Diseases, University of Bern, Bern, Switzerland

[3]Department of Infectious Diseases, Inselspital, Bern University Hospital, University of Bern, Bern, Switzerland

[4]Institute for Infectious Diseases, University of Bern, Bern, Switzerland

[5]Institute for Sensors and Electronics, University of Applied Sciences and Arts Northwestern Switzerland, Windisch, Switzerland

[6]Population Health Sciences, University of Bristol, Bristol, UK

[7]Centre for Infectious Disease Epidemiology and Research, University of Cape Town, Cape Town, South Africa

[8]Institute of Educational Science, University of Bern, Bern, Switzerland

[*]Corresponding author: lukas.fenner@ispm.unibe.ch

List of Texts

A Experimental determination of the CADR 4

B Data collection 6

C Epidemiological line list data 7

D Molecular line list data 9

E Modeling changes in particle concentrations 22

F Modeling relative risk of infection 23

F.1 Overall approach . . . 23

F.2 Notation . . . 23

F.3 Relating the number of respiratory cases to the number of infections . . . 23

F.4 Relating the number of new infections to the presence of interventions . . . 24

F.5 Specifying the distribution of the incubation period . . . 24

F.6 Adjusting for under-reporting of cases on weekends . . . 26

F.7 Modeling school-free days . . . 26

F.8 Seeding infections before study start . . . 27

F.9 Priors for modeling parameters . . . 27

G Modeling changes in cough frequency 28

H Detailed results for changes in particle concentrations 29

I Detailed results for risk of infection model 31

J Detailed results for cough frequency 34

K Possible transmission networks 35

References 37

List of Figures

S1 Measured concentration curves with and without air cleaner 5

S2 Choices of priors for the incubation periods 25

S3 Analysis of particle concentrations and comparison between study conditions 29

S4 Boxplot of environmental variables by study condition 30

S5 Comparison of model-estimates with observed case data 33

S6 Association between cough frequency and virus-specific positivity rate 34

S7 **Molecular detection of respiratory viruses and transmission network based on spatiotemporal analysis of students' saliva samples**. **(a)** Number of positive saliva samples by virus. **(b)** Daily number of positive saliva samples (colored circles) and possible transmission chains within classes (directed arrows). Positive samples are linked if they belong to the same virus and are less than 1 week apart. Positive samples from the air and filters as blank squares aligned. IFB: influenza B, HRV: human rhinovirus, AdV: adenovirus, CoV: SARS-CoV-2, MPV: human metapneumovirus, PIV: parainfluenza virus. 36

List of Tables

S1 Overview of collected data 6

S2 Line list of respiratory cases over the study period 7

S3 Line list of molecular test results over the study period 9

S4 Estimated reduction in aerosol and particle concentrations with interventions 30

S5 Estimation results from infection risk model 32

A Experimental determination of the CADR

The clean air delivery rate (CADR) is typically used to quantify the cleaning efficiency of air cleaners[1]. It is expressed in m^3/s and describes the volumetric flow rate of particle-free, clean air that an air cleaner delivers into the room. The CADR is equal to the product of the filtration efficiency and the volumetric flow rate passing through the device.

We followed the approach by Küpper et al. to determine the CADR[2]. The approach is based on the release of fine aerosols (particle diameter $D<1\mu$m) in a closed, unventilated room by measuring the decay rate k of the particle concentration $N(t)$ caused by the air cleaner, i. e.

$$N(t) = N_0 \cdot e^{-k \cdot t} \tag{1}$$

Since the surfaces in the room (e. g. walls and furniture) are also sinks for aerosol particles, measurements were carried out with (k_{cleaner}) and without air cleaners($k_{\text{background}}$). Based on that, we can compute the CADR as

$$\text{CADR} = V \cdot k_{\text{cleaner}} - V \cdot k_{\text{background}}, \tag{2}$$

where $V = 233 m^3$ is the volume of the classroom.

We ensured that the particle concentrations in the experiments were not too high, thereby preventing bias due to coagulation (i. e. the formation of a few large particles from many small particles). At the beginning of each experiment, fine aerosol particles were released for a few minutes by ultrasonic nebulization of an aqueous NaCl solution of 5 g/l. The generated dehydrated salt particles have a broad size distribution in the range of $0.02-1.0\mu$m. Decay rates were measured using 11 fine dust sensors (SPS30, Sensirion AG), which were placed at head height of the students. These sensors measured the number concentration of individual particles by counting the scattered light pulses they produce when passing through a laser beam. Because of their scattering properties, these sensors measure the number of particles larger than $\sim 0.3\,\mu$m.

Figure S1 shows the particle number concentration with and without without the air cleaner. The exponential decrease is clearly visible as a linear curve in the logarithmic plot. Based on these experiments, we were able to determine the net CADR values of the air cleaner as $420\,m^3/h$

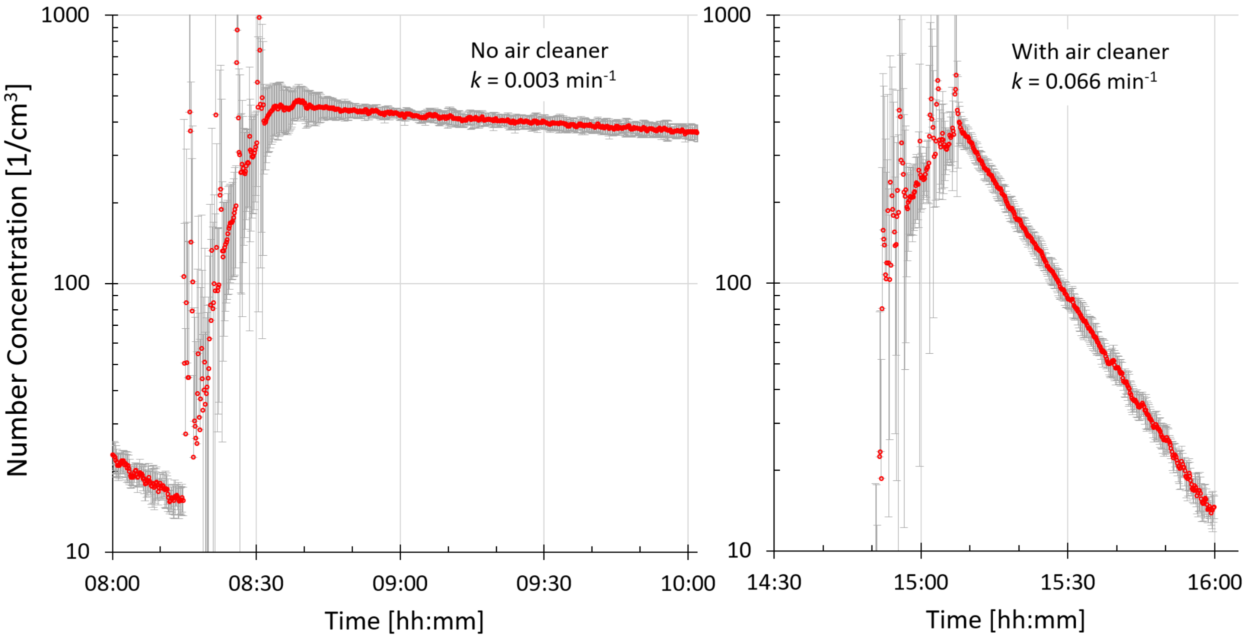

Figure S1. Measured concentration curves with and without air cleaner. The red dot shows the average concentration measured by the 11 distributed sensors of particles with $0.3\mu m < D < 1\mu m$. The standard deviation is shown in gray as an error bar. The increase in concentration at the beginning of these experiments is due to the release of the NaCl particles during approximately 15-20 min. The inhomogeneity of the concentration during the nebulization period is seen in larger error bars. Afterwards, the aerosol source is turned off, the concentration homogenizes quickly, and the concentration drop follows an exponential decay, which is used for the CADR calculation.

with an uncertainty of $\pm10\%$ due to systematic differences in the setup and measurement errors of the instruments. Our estimated CADR is lower than the CADR reported by the manufacturer of $600\text{m}^3/\text{h}$, most likely because the reported CADR was determined with comparably large particles (pollen in the super micrometer size range). Thus, we explain the lower filtration efficiency determined here with the fact that CADR is strongly dependent on the particle size, i. e. large particles are easier to filter than fine aerosols.

B Data collection

Table S1 in SI Appendixprovides an overview of the types of data that were collected in each classroom.

Table S1. Overview of collected data. Type of data collected, method/device, and frequency in the rooms of classes A and B.

Type of data	Method / device	Class A	Class B	Frequency
**Molecular data**				
Bioaerosol sampling	Bioaerosol sampling devices: BioSpot-VIVAS (Aerosol Devices Inc., Ft. Collins, Colorado, United States) and Coriolis Micro Air (Bertin Instruments Montigny-le/Bretonneux, France)	x	(x) only Coriolis	daily
Saliva samples	Saliva samples	x	x	twice per week
Swabs from air cleaners	Swab samples from Xiaomi HEPA filters (Xiaomi Mi Air Pro 70m2, Shenzhen, China)	x	x	after each intervention phase (both classes) and before the vacation (only class B)
**Environmental data**				
CO_2, aerosol/particle concentrations, humidity, temperature	Air quality device: AQ Guard (Palas GmbH, Karlsruhe, Germany)	x	x	daily by minute
Coughs	Sounds collected via microphones and coughs detected with a Machine Learning model[3]	x	x	daily by seconds
**Epidemiological data**				
Absences	Survey	x	x	daily
Student characteristics	Survey	x	x	at study start

C Epidemiological line list data

Reports about absences were entered electronically into a REDcap database[4,5] Table S2. shows the line list data for respiratory cases based on epidemiological data. For each class and case, it shows the date when the student was absent, s/he reported symptoms, and returned to school. We also collected data on laboratory tests but none of the students reported a test result during the study. A case of respiratory infection was defined as an absence where the student reported a sickness with at least one of the following symptoms: fever, coughing, tiredness, loss of test or smell, sore throat, headache, aches and pains, diarrhea, difficulty breathing or shortness of breath, stomach. Students were asked to report the first date they experienced symptoms, so that a student absent on Monday could report Saturday or Sunday as the day when symptoms began to be felt. We will refer respiratory cases by their dates of symptom onset, which usually corresponded to the absence date, unless the student attended the school while experiencing symptoms. Informed consent for one student could not be obtained and the single absence of this student was considered unrelated to a respiratory infection.

Table S2. Line list of respiratory cases over the study period.

Class	Date of absence	Date of symptom onset	Date of return
A	2023-01-16	2023-01-16	2023-01-19
A	2023-01-23	2023-01-23	2023-01-24
A	2023-02-05	2023-02-05	2023-02-13
A	2023-02-09	2023-02-09	2023-02-13
A	2023-02-17	2023-02-17	2023-02-20
A	2023-02-20	2023-02-20	2023-02-21
A	2023-02-20	2023-02-20	2023-02-23
A	2023-02-21	2023-02-21	2023-02-22
A	2023-02-27	2023-02-27	2023-02-28
A	2023-02-27	2023-02-27	2023-03-01
A	2023-02-25	2023-02-27	2023-03-06
A	2023-02-27	2023-02-28	2023-03-03
A	2023-03-06	2023-03-06	2023-03-07
A	2023-03-06	2023-03-06	2023-03-09
B	2023-01-16	2023-01-16	2023-01-17

B	2023-01-19	2023-01-19	2023-01-20
B	2023-01-16	2023-01-20	2023-01-23
B	2023-01-21	2023-01-23	2023-01-24
B	2023-01-23	2023-01-23	2023-01-24
B	2023-01-24	2023-01-24	2023-01-25
B	2023-02-01	2023-02-02	2023-02-13
B	2023-02-03	2023-02-03	2023-02-13
B	2023-02-17	2023-02-17	2023-02-21
B	2023-02-20	2023-02-20	2023-02-21
B	2023-02-20	2023-02-20	2023-02-28
B	2023-02-23	2023-02-23	2023-02-24
B	2023-02-27	2023-02-27	2023-03-01
B	2023-02-28	2023-02-28	2023-03-01
B	2023-02-28	2023-02-28	2023-03-01
B	2023-03-01	2023-03-01	2023-03-03
B	2023-03-01	2023-03-01	2023-03-06
B	2023-03-02	2023-03-02	2023-03-06
B	2023-03-03	2023-03-03	2023-03-08
B	2023-03-06	2023-03-06	2023-03-07
B	2023-03-08	2023-03-08	2023-03-09

D Molecular line list data

Table S3 shows the line list data for the laboratory test results from human saliva samples. For each class and test, it shows the date when the test was taken, the test result and (if positive) the virus that was detected. Recall that all students in class were sampled twice per week every Tuesday and Thursday.

Table S3. Line list of respiratory cases over the study period. IFB: Influenza B, HRV: rhinovirus, AdV: adenovirus, CoV: SARS-CoV-2, MPV: metapneumovirus, PIV: parainfluenza virus.

Class	Date of test	Weekday	Test result	Virus
A	2023-01-17	Tuesday	Negative	-
A	2023-01-17	Tuesday	Negative	-
A	2023-01-17	Tuesday	Negative	-
A	2023-01-17	Tuesday	Negative	-
A	2023-01-17	Tuesday	Negative	-
A	2023-01-17	Tuesday	Negative	-
A	2023-01-17	Tuesday	Negative	-
A	2023-01-17	Tuesday	Negative	-
A	2023-01-17	Tuesday	Negative	-
A	2023-01-17	Tuesday	Negative	-
A	2023-01-17	Tuesday	Negative	-
A	2023-01-17	Tuesday	Negative	-
A	2023-01-17	Tuesday	Negative	-
A	2023-01-17	Tuesday	Negative	-
A	2023-01-17	Tuesday	Negative	-
A	2023-01-17	Tuesday	Positive	AdV
A	2023-01-17	Tuesday	Positive	AdV
A	2023-01-19	Thursday	Negative	-
A	2023-01-19	Thursday	Negative	-
A	2023-01-19	Thursday	Negative	-
A	2023-01-19	Thursday	Negative	-
A	2023-01-19	Thursday	Negative	-
A	2023-01-19	Thursday	Negative	-
A	2023-01-19	Thursday	Negative	-
A	2023-01-19	Thursday	Negative	-
A	2023-01-19	Thursday	Negative	-

A	2023-01-19	Thursday	Negative	-
A	2023-01-19	Thursday	Negative	-
A	2023-01-19	Thursday	Negative	-
A	2023-01-19	Thursday	Negative	-
A	2023-01-19	Thursday	Negative	-
A	2023-01-19	Thursday	Positive	AdV
A	2023-01-19	Thursday	Positive	AdV
A	2023-01-24	Tuesday	Negative	-
A	2023-01-24	Tuesday	Negative	-
A	2023-01-24	Tuesday	Negative	-
A	2023-01-24	Tuesday	Negative	-
A	2023-01-24	Tuesday	Negative	-
A	2023-01-24	Tuesday	Negative	-
A	2023-01-24	Tuesday	Negative	-
A	2023-01-24	Tuesday	Negative	-
A	2023-01-24	Tuesday	Negative	-
A	2023-01-24	Tuesday	Negative	-
A	2023-01-24	Tuesday	Negative	-
A	2023-01-24	Tuesday	Negative	-
A	2023-01-24	Tuesday	Negative	-
A	2023-01-24	Tuesday	Negative	-
A	2023-01-24	Tuesday	Positive	AdV
A	2023-01-24	Tuesday	Positive	AdV
A	2023-01-24	Tuesday	Positive	HRV
A	2023-01-26	Thursday	Negative	-
A	2023-01-26	Thursday	Negative	-
A	2023-01-26	Thursday	Negative	-
A	2023-01-26	Thursday	Negative	-
A	2023-01-26	Thursday	Negative	-
A	2023-01-26	Thursday	Negative	-
A	2023-01-26	Thursday	Negative	-
A	2023-01-26	Thursday	Negative	-
A	2023-01-26	Thursday	Negative	-
A	2023-01-26	Thursday	Negative	-
A	2023-01-26	Thursday	Negative	-
A	2023-01-26	Thursday	Negative	-

A	2023-01-26	Thursday	Negative	-
A	2023-01-26	Thursday	Negative	-
A	2023-01-26	Thursday	Negative	-
A	2023-01-26	Thursday	Positive	AdV
A	2023-01-26	Thursday	Positive	AdV
A	2023-01-31	Tuesday	Negative	-
A	2023-01-31	Tuesday	Negative	-
A	2023-01-31	Tuesday	Negative	-
A	2023-01-31	Tuesday	Negative	-
A	2023-01-31	Tuesday	Negative	-
A	2023-01-31	Tuesday	Negative	-
A	2023-01-31	Tuesday	Negative	-
A	2023-01-31	Tuesday	Negative	-
A	2023-01-31	Tuesday	Negative	-
A	2023-01-31	Tuesday	Negative	-
A	2023-01-31	Tuesday	Negative	-
A	2023-01-31	Tuesday	Negative	-
A	2023-01-31	Tuesday	Negative	-
A	2023-01-31	Tuesday	Negative	-
A	2023-01-31	Tuesday	Negative	-
A	2023-01-31	Tuesday	Negative	-
A	2023-01-31	Tuesday	Negative	-
A	2023-01-31	Tuesday	Positive	AdV
A	2023-02-02	Thursday	Negative	-
A	2023-02-02	Thursday	Negative	-
A	2023-02-02	Thursday	Negative	-
A	2023-02-02	Thursday	Negative	-
A	2023-02-02	Thursday	Negative	-
A	2023-02-02	Thursday	Negative	-
A	2023-02-02	Thursday	Negative	-
A	2023-02-02	Thursday	Negative	-
A	2023-02-02	Thursday	Negative	-
A	2023-02-02	Thursday	Negative	-
A	2023-02-02	Thursday	Negative	-
A	2023-02-02	Thursday	Negative	-
A	2023-02-02	Thursday	Negative	-

A	2023-02-02	Thursday	Negative	-
A	2023-02-02	Thursday	Positive	AdV
A	2023-02-02	Thursday	Positive	AdV
A	2023-02-14	Tuesday	Negative	-
A	2023-02-14	Tuesday	Negative	-
A	2023-02-14	Tuesday	Negative	-
A	2023-02-14	Tuesday	Negative	-
A	2023-02-14	Tuesday	Negative	-
A	2023-02-14	Tuesday	Negative	-
A	2023-02-14	Tuesday	Negative	-
A	2023-02-14	Tuesday	Negative	-
A	2023-02-14	Tuesday	Negative	-
A	2023-02-14	Tuesday	Negative	-
A	2023-02-14	Tuesday	Negative	-
A	2023-02-14	Tuesday	Negative	-
A	2023-02-14	Tuesday	Negative	-
A	2023-02-14	Tuesday	Negative	-
A	2023-02-14	Tuesday	Negative	-
A	2023-02-14	Tuesday	Negative	-
A	2023-02-14	Tuesday	Negative	-
A	2023-02-14	Tuesday	Positive	MPV
A	2023-02-16	Thursday	Negative	-
A	2023-02-16	Thursday	Negative	-
A	2023-02-16	Thursday	Negative	-
A	2023-02-16	Thursday	Negative	-
A	2023-02-16	Thursday	Negative	-
A	2023-02-16	Thursday	Negative	-
A	2023-02-16	Thursday	Negative	-
A	2023-02-16	Thursday	Negative	-
A	2023-02-16	Thursday	Negative	-
A	2023-02-16	Thursday	Negative	-
A	2023-02-16	Thursday	Negative	-
A	2023-02-16	Thursday	Negative	-
A	2023-02-16	Thursday	Negative	-
A	2023-02-16	Thursday	Negative	-
A	2023-02-16	Thursday	Negative	-

A	2023-02-16	Thursday	Negative	-
A	2023-02-16	Thursday	Negative	-
A	2023-02-16	Thursday	Positive	MPV
A	2023-02-21	Tuesday	Negative	-
A	2023-02-21	Tuesday	Negative	-
A	2023-02-21	Tuesday	Negative	-
A	2023-02-21	Tuesday	Negative	-
A	2023-02-21	Tuesday	Negative	-
A	2023-02-21	Tuesday	Negative	-
A	2023-02-21	Tuesday	Negative	-
A	2023-02-21	Tuesday	Negative	-
A	2023-02-21	Tuesday	Negative	-
A	2023-02-21	Tuesday	Negative	-
A	2023-02-21	Tuesday	Negative	-
A	2023-02-21	Tuesday	Negative	-
A	2023-02-21	Tuesday	Negative	-
A	2023-02-21	Tuesday	Negative	-
A	2023-02-21	Tuesday	Negative	-
A	2023-02-21	Tuesday	Positive	CoV
A	2023-02-23	Thursday	Negative	-
A	2023-02-23	Thursday	Negative	-
A	2023-02-23	Thursday	Negative	-
A	2023-02-23	Thursday	Negative	-
A	2023-02-23	Thursday	Negative	-
A	2023-02-23	Thursday	Negative	-
A	2023-02-23	Thursday	Negative	-
A	2023-02-23	Thursday	Negative	-
A	2023-02-23	Thursday	Negative	-
A	2023-02-23	Thursday	Negative	-
A	2023-02-23	Thursday	Negative	-
A	2023-02-23	Thursday	Negative	-
A	2023-02-23	Thursday	Negative	-
A	2023-02-23	Thursday	Negative	-
A	2023-02-23	Thursday	Negative	-
A	2023-02-23	Thursday	Negative	-
A	2023-02-28	Tuesday	Negative	-

A	2023-02-28	Tuesday	Negative	-
A	2023-02-28	Tuesday	Negative	-
A	2023-02-28	Tuesday	Negative	-
A	2023-02-28	Tuesday	Negative	-
A	2023-02-28	Tuesday	Negative	-
A	2023-02-28	Tuesday	Negative	-
A	2023-02-28	Tuesday	Negative	-
A	2023-02-28	Tuesday	Negative	-
A	2023-02-28	Tuesday	Negative	-
A	2023-02-28	Tuesday	Negative	-
A	2023-02-28	Tuesday	Negative	-
A	2023-02-28	Tuesday	Negative	-
A	2023-02-28	Tuesday	Positive	IFB
A	2023-03-02	Thursday	Negative	-
A	2023-03-02	Thursday	Negative	-
A	2023-03-02	Thursday	Negative	-
A	2023-03-02	Thursday	Negative	-
A	2023-03-02	Thursday	Negative	-
A	2023-03-02	Thursday	Negative	-
A	2023-03-02	Thursday	Negative	-
A	2023-03-02	Thursday	Negative	-
A	2023-03-02	Thursday	Negative	-
A	2023-03-02	Thursday	Negative	-
A	2023-03-02	Thursday	Negative	-
A	2023-03-02	Thursday	Negative	-
A	2023-03-02	Thursday	Negative	-
A	2023-03-02	Thursday	Negative	-
A	2023-03-02	Thursday	Positive	AdV
A	2023-03-07	Tuesday	Negative	-
A	2023-03-07	Tuesday	Negative	-
A	2023-03-07	Tuesday	Negative	-
A	2023-03-07	Tuesday	Negative	-
A	2023-03-07	Tuesday	Negative	-
A	2023-03-07	Tuesday	Negative	-
A	2023-03-07	Tuesday	Negative	-
A	2023-03-07	Tuesday	Negative	-

A	2023-03-07	Tuesday	Negative	-
A	2023-03-07	Tuesday	Negative	-
A	2023-03-07	Tuesday	Negative	-
A	2023-03-07	Tuesday	Negative	-
A	2023-03-07	Tuesday	Negative	-
A	2023-03-07	Tuesday	Negative	-
A	2023-03-07	Tuesday	Negative	-
A	2023-03-07	Tuesday	Positive	HRV
A	2023-03-07	Tuesday	Positive	HRV
A	2023-03-09	Thursday	Negative	-
A	2023-03-09	Thursday	Negative	-
A	2023-03-09	Thursday	Negative	-
A	2023-03-09	Thursday	Negative	-
A	2023-03-09	Thursday	Negative	-
A	2023-03-09	Thursday	Negative	-
A	2023-03-09	Thursday	Negative	-
A	2023-03-09	Thursday	Negative	-
A	2023-03-09	Thursday	Negative	-
A	2023-03-09	Thursday	Negative	-
A	2023-03-09	Thursday	Negative	-
A	2023-03-09	Thursday	Negative	-
A	2023-03-09	Thursday	Negative	-
A	2023-03-09	Thursday	Positive	HRV
A	2023-03-09	Thursday	Positive	HRV
A	2023-03-09	Thursday	Positive	HRV
A	2023-03-09	Thursday	Positive	IFB
B	2023-01-17	Tuesday	Negative	-
B	2023-01-17	Tuesday	Negative	-
B	2023-01-17	Tuesday	Negative	-
B	2023-01-17	Tuesday	Negative	-
B	2023-01-17	Tuesday	Negative	-
B	2023-01-17	Tuesday	Negative	-
B	2023-01-17	Tuesday	Negative	-
B	2023-01-17	Tuesday	Negative	-
B	2023-01-17	Tuesday	Negative	-
B	2023-01-17	Tuesday	Negative	-

B	2023-01-17	Tuesday	Negative	-
B	2023-01-17	Tuesday	Negative	-
B	2023-01-17	Tuesday	Positive	HRV
B	2023-01-17	Tuesday	Positive	HRV
B	2023-01-17	Tuesday	Positive	IFB
B	2023-01-19	Thursday	Negative	-
B	2023-01-19	Thursday	Negative	-
B	2023-01-19	Thursday	Negative	-
B	2023-01-19	Thursday	Negative	-
B	2023-01-19	Thursday	Negative	-
B	2023-01-19	Thursday	Negative	-
B	2023-01-19	Thursday	Negative	-
B	2023-01-19	Thursday	Negative	-
B	2023-01-19	Thursday	Negative	-
B	2023-01-19	Thursday	Negative	-
B	2023-01-19	Thursday	Negative	-
B	2023-01-19	Thursday	Negative	-
B	2023-01-19	Thursday	Negative	-
B	2023-01-19	Thursday	Negative	-
B	2023-01-19	Thursday	Negative	-
B	2023-01-19	Thursday	Positive	IFB
B	2023-01-24	Tuesday	Negative	-
B	2023-01-24	Tuesday	Negative	-
B	2023-01-24	Tuesday	Negative	-
B	2023-01-24	Tuesday	Negative	-
B	2023-01-24	Tuesday	Negative	-
B	2023-01-24	Tuesday	Negative	-
B	2023-01-24	Tuesday	Negative	-
B	2023-01-24	Tuesday	Negative	-
B	2023-01-24	Tuesday	Negative	-
B	2023-01-24	Tuesday	Negative	-
B	2023-01-24	Tuesday	Negative	-
B	2023-01-24	Tuesday	Negative	-
B	2023-01-24	Tuesday	Positive	HRV
B	2023-01-26	Thursday	Negative	-
B	2023-01-26	Thursday	Negative	-

B	2023-01-26	Thursday	Negative	-
B	2023-01-26	Thursday	Negative	-
B	2023-01-26	Thursday	Negative	-
B	2023-01-26	Thursday	Negative	-
B	2023-01-26	Thursday	Negative	-
B	2023-01-26	Thursday	Negative	-
B	2023-01-26	Thursday	Negative	-
B	2023-01-26	Thursday	Negative	-
B	2023-01-26	Thursday	Negative	-
B	2023-01-26	Thursday	Negative	-
B	2023-01-26	Thursday	Positive	IFB
B	2023-01-26	Thursday	Positive	IFB
B	2023-01-26	Thursday	Positive	PIV
B	2023-01-31	Tuesday	Negative	-
B	2023-01-31	Tuesday	Negative	-
B	2023-01-31	Tuesday	Negative	-
B	2023-01-31	Tuesday	Negative	-
B	2023-01-31	Tuesday	Negative	-
B	2023-01-31	Tuesday	Negative	-
B	2023-01-31	Tuesday	Negative	-
B	2023-01-31	Tuesday	Negative	-
B	2023-01-31	Tuesday	Negative	-
B	2023-01-31	Tuesday	Negative	-
B	2023-01-31	Tuesday	Negative	-
B	2023-01-31	Tuesday	Negative	-
B	2023-01-31	Tuesday	Negative	-
B	2023-01-31	Tuesday	Negative	-
B	2023-01-31	Tuesday	Positive	IFB
B	2023-01-31	Tuesday	Positive	IFB
B	2023-02-02	Thursday	Negative	-
B	2023-02-02	Thursday	Negative	-
B	2023-02-02	Thursday	Negative	-
B	2023-02-02	Thursday	Negative	-
B	2023-02-02	Thursday	Negative	-
B	2023-02-02	Thursday	Negative	-
B	2023-02-02	Thursday	Negative	-

B	2023-02-02	Thursday	Negative	-
B	2023-02-02	Thursday	Negative	-
B	2023-02-02	Thursday	Negative	-
B	2023-02-02	Thursday	Negative	-
B	2023-02-02	Thursday	Negative	-
B	2023-02-02	Thursday	Negative	-
B	2023-02-02	Thursday	Negative	-
B	2023-02-02	Thursday	Negative	-
B	2023-02-02	Thursday	Positive	IFB
B	2023-02-14	Tuesday	Negative	-
B	2023-02-14	Tuesday	Negative	-
B	2023-02-14	Tuesday	Negative	-
B	2023-02-14	Tuesday	Negative	-
B	2023-02-14	Tuesday	Negative	-
B	2023-02-14	Tuesday	Negative	-
B	2023-02-14	Tuesday	Negative	-
B	2023-02-14	Tuesday	Negative	-
B	2023-02-14	Tuesday	Negative	-
B	2023-02-14	Tuesday	Negative	-
B	2023-02-14	Tuesday	Negative	-
B	2023-02-14	Tuesday	Negative	-
B	2023-02-14	Tuesday	Negative	-
B	2023-02-14	Tuesday	Negative	-
B	2023-02-14	Tuesday	Negative	-
B	2023-02-14	Tuesday	Negative	-
B	2023-02-14	Tuesday	Negative	-
B	2023-02-14	Tuesday	Positive	IFB
B	2023-02-16	Thursday	Negative	-
B	2023-02-16	Thursday	Negative	-
B	2023-02-16	Thursday	Negative	-
B	2023-02-16	Thursday	Negative	-
B	2023-02-16	Thursday	Negative	-
B	2023-02-16	Thursday	Negative	-
B	2023-02-16	Thursday	Negative	-
B	2023-02-16	Thursday	Negative	-
B	2023-02-16	Thursday	Negative	-

B	2023-02-16	Thursday	Negative	-
B	2023-02-16	Thursday	Negative	-
B	2023-02-16	Thursday	Negative	-
B	2023-02-16	Thursday	Negative	-
B	2023-02-16	Thursday	Negative	-
B	2023-02-16	Thursday	Negative	-
B	2023-02-16	Thursday	Negative	-
B	2023-02-16	Thursday	Negative	-
B	2023-02-16	Thursday	Negative	-
B	2023-02-21	Tuesday	Negative	-
B	2023-02-21	Tuesday	Negative	-
B	2023-02-21	Tuesday	Negative	-
B	2023-02-21	Tuesday	Negative	-
B	2023-02-21	Tuesday	Negative	-
B	2023-02-21	Tuesday	Negative	-
B	2023-02-21	Tuesday	Negative	-
B	2023-02-21	Tuesday	Negative	-
B	2023-02-21	Tuesday	Negative	-
B	2023-02-21	Tuesday	Negative	-
B	2023-02-21	Tuesday	Negative	-
B	2023-02-21	Tuesday	Negative	-
B	2023-02-21	Tuesday	Negative	-
B	2023-02-21	Tuesday	Negative	-
B	2023-02-21	Tuesday	Positive	CoV
B	2023-02-21	Tuesday	Positive	HRV
B	2023-02-23	Thursday	Negative	-
B	2023-02-23	Thursday	Negative	-
B	2023-02-23	Thursday	Negative	-
B	2023-02-23	Thursday	Negative	-
B	2023-02-23	Thursday	Negative	-
B	2023-02-23	Thursday	Negative	-
B	2023-02-23	Thursday	Negative	-
B	2023-02-23	Thursday	Negative	-
B	2023-02-23	Thursday	Negative	-
B	2023-02-23	Thursday	Negative	-
B	2023-02-23	Thursday	Negative	-

B	2023-02-23	Thursday	Negative	-
B	2023-02-23	Thursday	Positive	CoV
B	2023-02-23	Thursday	Positive	HRV
B	2023-02-28	Tuesday	Negative	-
B	2023-02-28	Tuesday	Negative	-
B	2023-02-28	Tuesday	Negative	-
B	2023-02-28	Tuesday	Negative	-
B	2023-02-28	Tuesday	Negative	-
B	2023-02-28	Tuesday	Negative	-
B	2023-02-28	Tuesday	Negative	-
B	2023-02-28	Tuesday	Negative	-
B	2023-02-28	Tuesday	Negative	-
B	2023-02-28	Tuesday	Negative	-
B	2023-02-28	Tuesday	Negative	-
B	2023-02-28	Tuesday	Positive	HRV
B	2023-02-28	Tuesday	Positive	IFB
B	2023-03-02	Thursday	Negative	-
B	2023-03-02	Thursday	Negative	-
B	2023-03-02	Thursday	Negative	-
B	2023-03-02	Thursday	Negative	-
B	2023-03-02	Thursday	Negative	-
B	2023-03-02	Thursday	Negative	-
B	2023-03-02	Thursday	Negative	-
B	2023-03-02	Thursday	Negative	-
B	2023-03-02	Thursday	Negative	-
B	2023-03-02	Thursday	Negative	-
B	2023-03-02	Thursday	Negative	-
B	2023-03-02	Thursday	Positive	IFB
B	2023-03-07	Tuesday	Negative	-
B	2023-03-07	Tuesday	Negative	-
B	2023-03-07	Tuesday	Negative	-
B	2023-03-07	Tuesday	Negative	-
B	2023-03-07	Tuesday	Negative	-
B	2023-03-07	Tuesday	Negative	-
B	2023-03-07	Tuesday	Negative	-
B	2023-03-07	Tuesday	Negative	-

B	2023-03-07	Tuesday	Negative	-
B	2023-03-07	Tuesday	Negative	-
B	2023-03-07	Tuesday	Negative	-
B	2023-03-07	Tuesday	Negative	-
B	2023-03-07	Tuesday	Negative	-
B	2023-03-07	Tuesday	Positive	AdV
B	2023-03-07	Tuesday	Positive	HRV
B	2023-03-07	Tuesday	Positive	IFB
B	2023-03-07	Tuesday	Positive	IFB
B	2023-03-09	Thursday	Negative	-
B	2023-03-09	Thursday	Negative	-
B	2023-03-09	Thursday	Negative	-
B	2023-03-09	Thursday	Negative	-
B	2023-03-09	Thursday	Negative	-
B	2023-03-09	Thursday	Negative	-
B	2023-03-09	Thursday	Negative	-
B	2023-03-09	Thursday	Negative	-
B	2023-03-09	Thursday	Negative	-
B	2023-03-09	Thursday	Negative	-
B	2023-03-09	Thursday	Negative	-
B	2023-03-09	Thursday	Negative	-
B	2023-03-09	Thursday	Negative	-
B	2023-03-09	Thursday	Positive	AdV
B	2023-03-09	Thursday	Positive	HRV
B	2023-03-09	Thursday	Positive	HRV
B	2023-03-09	Thursday	Positive	IFB

E Modeling changes in particle concentrations

We only analyze particle concentration data of the time periods during which students were in the classroom. Particle concentrations Y on day t in class j are summarized daily with the mean across these time periods. The change in aerosol number and particle matter mass concentrations is then estimated using Bayesian log-linear regression models

$$
\begin{aligned}
\log Y|\beta_0,\beta_1,\ldots,\beta_6 &\sim \text{Normal}(\mu,\sigma) \\
\mu &= \beta_0 + \beta_1 \cdot \text{AirCleaner} + \beta_2 \cdot \text{Class} + \beta_3 \cdot \text{Weekday} + \beta_4 \cdot \text{Students} \\
&\quad + \beta_5 \cdot \text{AirChangeRate} + \beta_6 \cdot \text{Cases} \\
\beta_0 &\sim \text{Student-t}_5(\mu_y, 2.5 s_y) \\
\beta_1,\ldots,\beta_6 &\sim \text{Student-t}_5\left(0, 2.5\frac{s_y}{s_x}\right) \\
\sigma &\sim \text{Exponential}\left(\frac{1}{s_y}\right) .
\end{aligned}
$$

The effect of air cleaners is adjusted for class- and weekday-specific effects, the number of students in school, the air change rate, and the cumulative number of cases related to respiratory infections. A log transform is applied to all continuous input variables.

F Modeling relative risk of infection

F.1 Overall approach

The overall aim is to estimate the effects of air cleaners on the daily number of respiratory infections. The latter is unobserved/latent and inferred from the daily number of respiratory cases (absences related to respiratory infections by date of symptom onset), considering the delay from infection to symptom onset (i. e. the incubation period). The effect of air cleaners is estimated with a Bayesian approach, which requires the specification of prior distributions for all model parameters.

F.2 Notation

j	class
t	days since start of study period
C_{jt}	number of new cases among students in class j at day t (observed)
I_{jt}	number of new infections in class j at day t (unobserved)
F_{jt}	number of new infections in class j before day t (unobserved)
N_{jt}	cumulative number of infections in class j until day t (unobserved)

F.3 Relating the number of respiratory cases to the number of infections

The number of respiratory cases C in class j at time t is modeled with a Negative Binomial distribution

$$C_{jt} \sim \text{Negative-Binomial}(\mu_{jt}, \phi),$$

where μ_{jt} is the expected number of new cases and ϕ is the parameter modeling over-dispersion. The expected number of new cases is the weighted sum of the number of new infections I_{jt} in the previous days

$$\mu_{jt} = \sum_{s<t} I_{js} \cdot p_{\text{IN}}(t-s),$$

where $p_{\mathrm{IN}}(t-s)$ denotes the probability distribution of the incubation period.

F.4 Relating the number of new infections to the presence of interventions

The number of new infections is related to the presence of air cleaners using a log-link

$$\log I_{jt} = \log F_{jt} - \log N_{jt} + \beta_0 + \beta_1 \cdot \mathrm{AirCleaner}_{jt} + \beta_2 \cdot \mathrm{Class}_t + \beta_3 \cdot \mathrm{Students}_{jt}$$
$$+ \beta_4 \cdot \mathrm{AirChangeRate}_{jt} + \beta_5 \cdot \mathrm{CoV}_t + \beta_6 \cdot \mathrm{ILI}_t,$$

where $F_{jt} = \sum_{t-7}^{t-1} I_{js}$ is the number of infections in the previous seven days (first model offset; a proxy for the number of contagious students), $N_{jt} = \sum_{s<t} I_{js}$ is the cumulative number of infections (second model offset; the inverse is a proxy for the number of susceptible students), β_0 is the rate of new infections without air cleaners (model intercept), and β_1 is the effect of air cleaners. The effect of air cleaners is adjusted for class-specific effects, the number of students in school, the air change rate, the proportion of positive tests for SARS-CoV-2 in the community, and the number of consultations for influenza-like illnesses in the community.

Note that we deviated from the statistical analysis plan by using as model offset $\log F_{jt} - \log N_{jt}$ instead of just $\log N_{jt}$. This change improved the fit of our model. The reason is that the ratio $\log(F_{jt}/N_{jt})$ incorporates both ways by which new infections can naturally decrease over time, i. e. a decrease in contagious students (F_{jt}) or a decrease in susceptible students ($1/N_{jt}$).

F.5 Specifying the distribution of the incubation period

The virus of each respiratory infection could not be identified from the epidemiological data because the students never obtained a laboratory test result. As a consequence, different incubation periods need to be considered in p_{IN}, reflecting a combination of the virus-specific incubation periods. The combination is determined based on the weekly proportion of positive saliva samples for each virus found in the molecular analysis. Formally, let p_{IN}^v be the distribution for the incubation period of respiratory virus v and let pp_{vw} be the proportion of positive saliva samples in study week w, then each week the combined incubation period is computed as the weighted sum of the virus-specific

incubation periods

$$p^w_{\mathrm{IN}}(s) = \sum_v p^v_{\mathrm{IN}}(s) \cdot pp_{vw} \quad \forall s. \tag{3}$$

The prior distributions for the virus-specific incubation periods are based on estimates published in the literature[6,7]. The distributions are shown in Figure S2. Since we could not obtain prior estimates for the incubation period of metapneumovirus (MPV) from the literature, we instead formed a distribution by using the equally weighted average of the parameters from the other distributions.

We estimate the virus-specific distributions from our data as part of fitting the overall model. Furthermore, note that p_{IN} is discretized via $p_{\mathrm{IN}}(s) = \int_0^{0.5} p_{\mathrm{IN}}(\tau)\,\mathrm{d}\tau$ for $s = 0$ and $p_{\mathrm{IN}}(s) = \int_{s-0.5}^{s+0.5} p_{\mathrm{IN}}(\tau)\,\mathrm{d}\tau$ for $s > 0$, where $p_{\mathrm{IN}}(\tau) \sim \mathrm{Lognormal}(\mu, \sigma)$ is the density of the lognormal distribution.

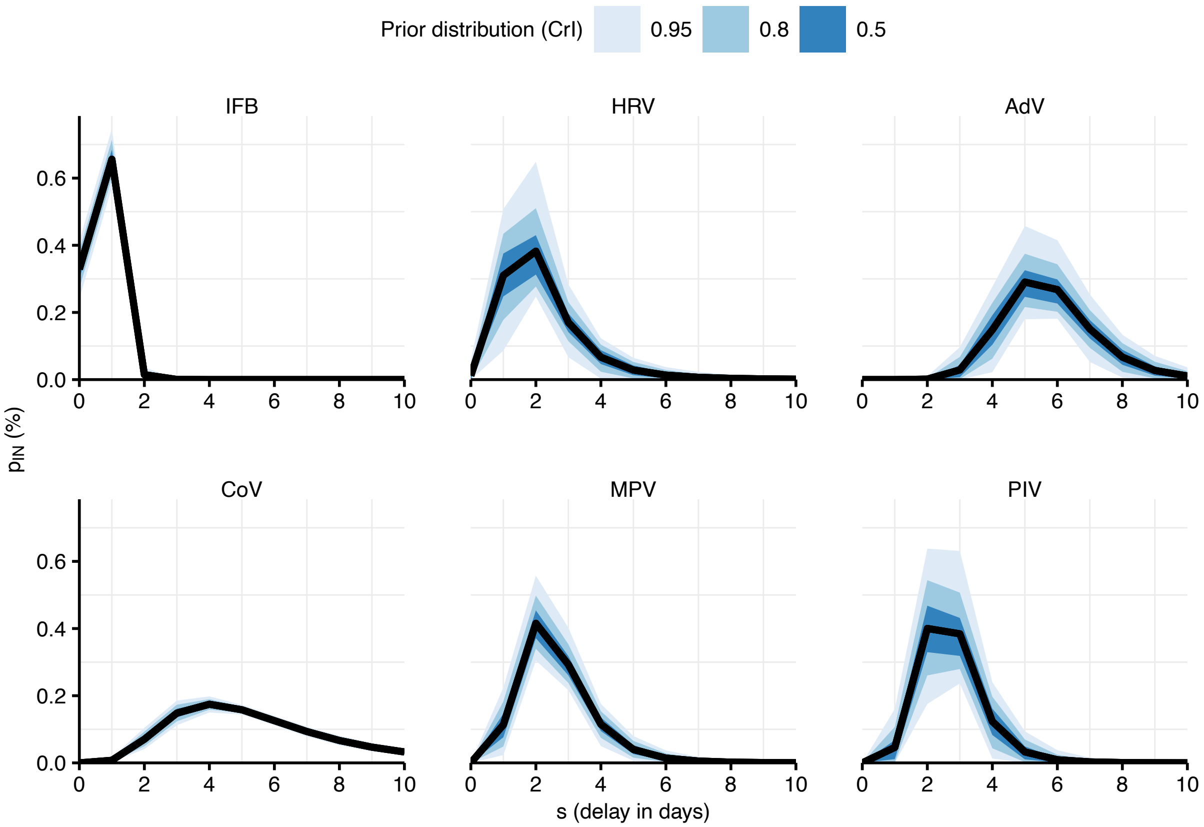

Figure S2. Choices of priors for the distribution of the virus-specific incubation periods. IFB: Influenza B, HRV: rhinovirus, AdV: adenovirus, CoV: SARS-CoV-2, MPV: metapneumovirus, PIV: parainfluenza virus.

F.6 Adjusting for under-reporting of cases on weekends

Despite recording cases by date of symptom onset, we recorded a higher proportion of cases on Mondays than on weekends, suggesting recall bias and under-reporting of cases on weekends. To consider weekday effects in the reporting of cases, we re-weight the expected number of cases each week as follows. Let $k \in$(1: Saturday, 2: Sunday, 3: Monday, ..., 7: Friday) denote the weekday with the week starting on Saturday. The re-weighted expected number of cases $\tilde{\mu}$ (class and day indexes omitted) are computed as

$$\tilde{\mu}_k = \mu_k \cdot \vartheta_k \cdot \left(\frac{\sum_k \mu_k}{\sum_k \nu_k \cdot \mu_k} \right)$$
$$\sum_k \vartheta_k = 1,$$

where ν_k is the weight for weekday k. These weights are modeled with a Dirichlet prior

$$\vartheta \sim \text{Dirichlet}(c)$$
$$c_k = \sum_j \sum_t C_{jt} \mathbb{I}_{\text{Weekday}(t)=k} \quad \forall k$$

where c_k is the total number of cases reported for weekday k and $\mathbb{I}$ is a binary indicator.

F.7 Modeling school-free days

Infections may have occurred during the week of vacation that falls into the study period. The expected number of infections and cases are computed during vacation, but vacation days are not modeled (i. e. not incorporated into the model likelihood). In addition, we assume lower transmission of respiratory infections on days without school (weekends and vacations). We incorporate our prior belief into the model intercept β_0

$$\beta_0 = \alpha + \omega \cdot \text{NoSchool},$$

where α is the rate of new infections on school days and $\alpha + \omega$ is the rate on days without school. We model ω with an informative prior for a 10% decrease in new infections on school-free days

$$\omega \sim \text{Normal}(\log 1.1, 0.05) \ .$$

F.8 Seeding infections before study start

Cases in the first week of the study could indicate infections before the study commenced. We will therefore seed our model $2 \cdot \mathrm{m}$ days before the study start, where m is the average incubation period of the virus with the largest incubation period (i. e. adenovirus). The number of infections before the study start will be modeled with an exponential prior

$$I_{jt} \sim \text{Exponential}(1) \quad t = -2 \cdot \mathrm{m} + 1, \ldots, 0 \ .$$

Note that we deviated from the statistical analysis plan by changing the parameter of the Exponential distribution from $\lambda = 2 \cdot m$ to $\lambda = 1$. After inspecting the model fit, we realized that the model could not adequately fit the number of new cases at the start of study, because the expected number of new infections in the seeding period $\mu = 1/\lambda = 1/(2 \cdot m)$ were too low. After increasing the expected number of new infections in the seeding period to $\mu = 1$ the model could adequately capture new respiratory cases observed at the study start.

F.9 Priors for modeling parameters

We use weakly informative priors for all modeling parameters following recommendations on the choice of priors[8–12]. The continuous adjustment variables are standardized to have zero mean and a standard deviation of 0.5.

$$\begin{aligned} \alpha &\sim \text{Student-t}_5(0, 10) \\ \beta_2, \ldots, \beta_6 &\sim \text{Student-t}_5(0, 2.5) \\ \frac{1}{\sqrt{\phi}} &\sim \text{Half-Normal}(0, 1), \end{aligned}$$

where Student-t_5 is a Student-t distribution with 5 degrees of freedom.

G Modeling changes in cough frequency

We analyze the daily number of coughs Y with a Negative Binomial regression model

$$
\begin{aligned}
Y|\beta_0, \beta_1, \ldots, \beta_6 &\sim \text{Negative-Binomial}(\mu, \phi) \\
\log \mu &= \log T + \beta_0 + \beta_1 \cdot \text{AirCleaner} + \beta_2 \cdot \text{Class} + \beta_3 \cdot \text{Weekday} + \beta_4 \cdot \text{Students} + \beta_5 \cdot \text{Ventilation} + \beta_6 \cdot \\
\beta_0 &\sim \text{Student-t}_5(0, 2.5) \\
\beta_1, \ldots, \beta_6 &\sim \text{Student-t}_5\left(0, \frac{2.5}{s_x}\right) \\
\frac{1}{\sqrt{\phi}} &\sim \text{Half-Normal}(0, 1),
\end{aligned}
$$

where T is the daily duration that students were in the classroom and ϕ is the parameter modeling over-dispersion in count data.

In addition, we analyze the association between the daily number of coughs Y and the number of positive saliva test resutls for respiratory viruses with a Negative Binomial hierarchical regression model

$$
\begin{aligned}
Y|\beta_0, \theta_{[v]}, \theta, \tau &\sim \text{Negative-Binomial}(\mu, \phi) \\
\log \mu &= \log T + \beta_0 + \sum_v \theta_{[v]} \cdot \text{Saliva}_v \\
\beta_0 &\sim \text{Student-t}_5(0, 2.5) \\
\theta_{[\text{IFB}]}, \ldots, \theta_{[\text{PIV}]} &\sim \text{Normal}\,(\theta, \tau) \\
\theta &\sim \text{Student-t}_5\left(0, \frac{2.5}{s_v}\right) \\
\tau &\sim \text{Half-Normal}\,(0, 1) \\
\frac{1}{\sqrt{\phi}} &\sim \text{Half-Normal}(0, 1),
\end{aligned}
$$

where $\theta_{[v]}$ is the partially pooled estimate for respiratory virus $v \in$ (IFB, HRV, AdV, CoV, MPV, PIV), τ estimates variation between viruses, and s_v is the average standard deviation across the count variables of the respiratory viruses.

H Detailed results for changes in particle concentrations

There was a strong difference in particle concentrations between study conditions (Figure S3a). When adjusting for air change rates and multiple other factors, the aerosol number concentration decreased by 76% (95%-CrI 63% to 86%) with air cleaners (Figure S3b and Table S4). The decrease in the concentration of larger particles (PM_{10}) was greater than the decrease in the concentration of smaller particles (PM_1 to PM_4).

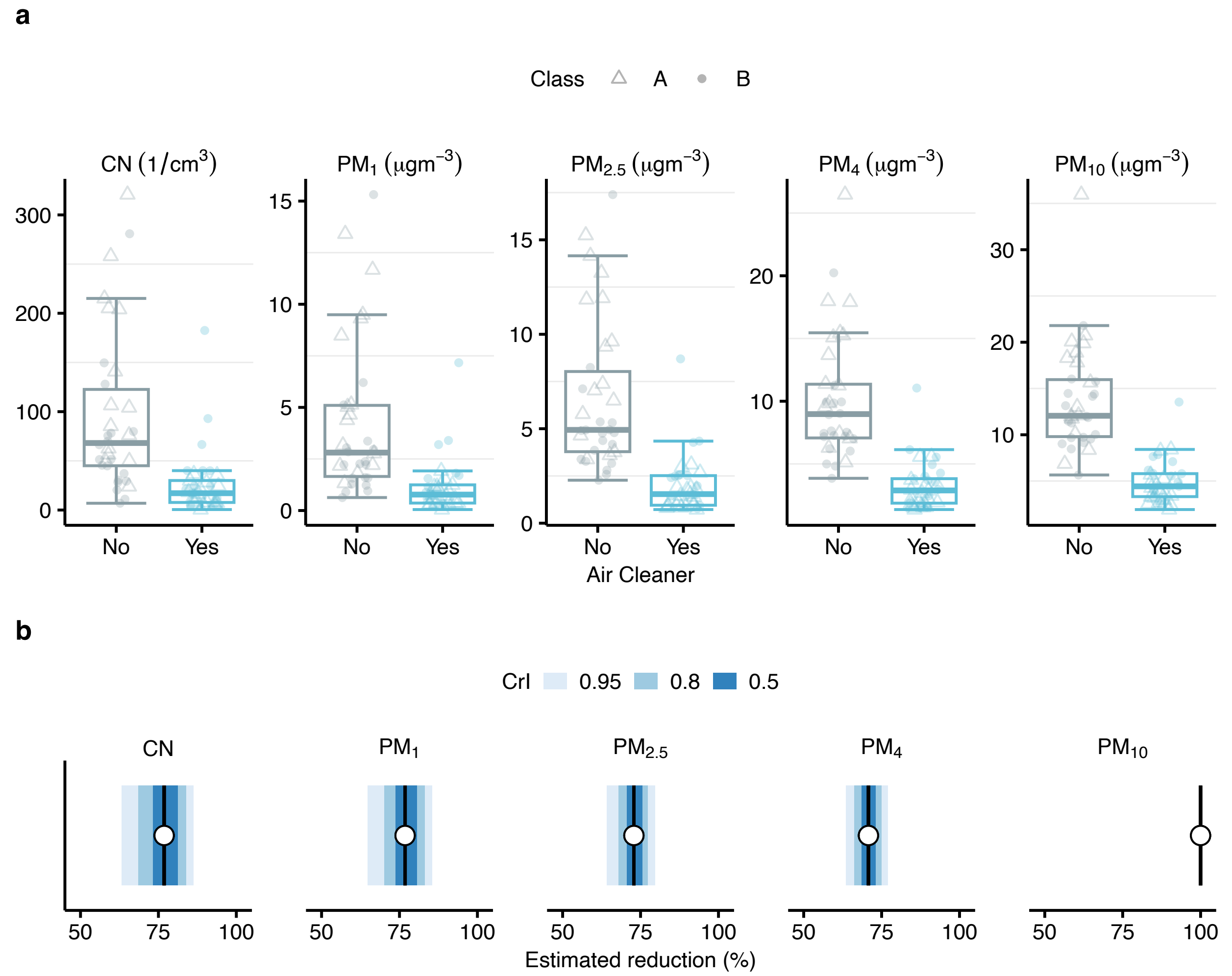

Figure S3. Analysis of particle concentrations and comparison between study conditions. (a) Boxplot of the daily average values for aerosol number concentration (CN in $1/cm^3$) and particle mass concentration (PM for particles of sizes <1 to <10 μm, respectively in μgm^{-3}). **(b)** Estimated reduction in aerosol number and particle mass concentrations with air cleaners (posterior mean as dot and 50%-, 80%- and 95%-CrI as lines, respectively).

In the main paper, we compared particle concentrations between study conditions. Figure S4

Table S4. Estimated reduction in aerosol number (CN) and particle mass (PM) concentrations with interventions (posterior mean and upper and lower estimate from the 95%-CrI).

Variable	Mean	Lower	Upper
CN	76.83	63.20	86.28
PM_1	76.79	64.84	85.46
$PM_{2.5}$	72.86	64.24	79.66
PM_4	70.77	63.46	76.97
PM_{10}	99.99	99.94	100.00

compares additional environmental variables, i.e. CO_2, air change rate, relative humidity, and temperature. The air change rate was estimated from indoor CO_2 levels using a transient mass balance model[13]. There are no considerable differences between in any of the variables between study conditions. Furthermore, numerical estimation results for the reduction in particle concentrations with air cleaners are shown in Table S4.

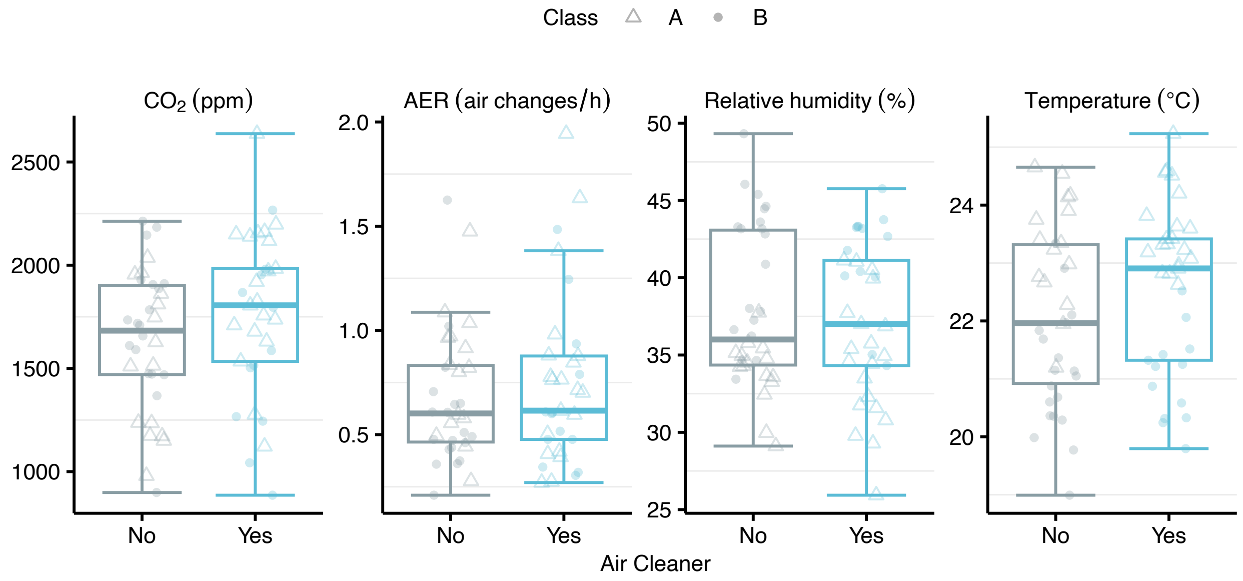

Figure S4. Boxplot for the daily average values of each environmental variable by study condition.

I Detailed results for risk of infection model

Table S5 presents the posterior mean, credible intervals and model diagnostics for all model parameters. See the main paper for a discussion of the effects of interventions. Here we briefly discuss some of the additional model parameters.

The effective sample size (ESS) and the Gelman-Rubin convergence diagnostic ($\hat{R}$) indicate good estimation power. It further suggests that the Markov chains converged.

The estimate for β_2 is positive, suggesting a higher risk of infection in class B, which is in line with the higher number of cases in this class. The estimate for β_3 is positive, suggesting a higher risk of infection as more students were in class. The estimate for β_4 is negative, suggesting a lower risk of infection with higher air change rates, in line with the intuition that the risk of infection is lower when the classroom is better ventilated. The estimate for β_5 and β_6 are both positive although only the former is distinguishable from zero, suggesting that higher transmission in the community was also associated with a higher risk of infection in the school.

The overdispersion parameter (ϕ) cannot be precisely estimated, but the credible interval suggests rather small overdispersion ($\phi \to \infty$). The school-free effect ω, the weekday weights ϑ, and the parameters for the distribution of the incubation period $\mu_{p_{\text{IN}}}$ and $\sigma_{p_{\text{IN}}}$ are all close to their priors, suggest that these parameters are barely informed by date but more by our prior assumptions.

Our model accounts for the delay from infection to case confirmation and allows transmission to change only at the dates of interventions. It thus reflects overall trends in transmission during study conditions rather than day-to-day variation. To evaluate how well our model fits these trends, Figure S5 compares the model-estimated expected number of cases with the observed number cases only on a weekly basis and across classes. Overall the estimates are in relatively good agreement. The 95%-CrIs of the model-estimates always include the observed cases.

Table S5. Estimation results from infection risk model for the number of new respiratory cases.

Parameter	Mean	Lower 95%-CrI	Upper 95%-CrI	$\hat{R}$	ESS
ϕ	4785.34	0.68	2733.84	1.00	2675
α	−0.59	−1.54	0.26	1.00	2457
ω	0.10	0.00	0.19	1.00	10312
β_1	−0.32	−0.81	0.17	1.00	2613
β_2	0.92	−0.04	1.92	1.00	2809
β_3	1.07	0.56	1.72	1.00	2183
β_4	−1.40	−2.70	−0.22	1.00	1743
β_5	0.81	0.48	1.15	1.00	2006
β_6	0.10	−0.20	0.50	1.00	2089
$\vartheta_{\text{Saturday}}$	0.05	0.01	0.12	1.00	7401
$\vartheta_{\text{Sunday}}$	0.03	0.00	0.08	1.00	7481
$\vartheta_{\text{Monday}}$	0.47	0.34	0.60	1.00	7909
$\vartheta_{\text{Tuesday}}$	0.13	0.06	0.22	1.00	9220
$\vartheta_{\text{Wednesday}}$	0.12	0.05	0.21	1.00	6220
$\vartheta_{\text{Thursday}}$	0.09	0.04	0.18	1.00	6539
$\vartheta_{\text{Friday}}$	0.11	0.05	0.20	1.00	6028
$\mu_{p_{\text{IN}}^{\text{IFB}}}$	−0.51	−0.60	−0.42	1.00	8269
$\mu_{p_{\text{IN}}^{\text{HRV}}}$	0.76	0.50	1.02	1.00	7381
$\mu_{p_{\text{IN}}^{\text{AdV}}}$	1.72	1.59	1.87	1.00	7370
$\mu_{p_{\text{IN}}^{\text{CoV}}}$	1.63	1.51	1.75	1.00	9076
$\mu_{p_{\text{IN}}^{\text{MPV}}}$	0.89	0.73	1.05	1.00	7591
$\mu_{p_{\text{IN}}^{\text{PIV}}}$	0.96	0.76	1.16	1.00	10017
$\sigma_{p_{\text{IN}}^{\text{IFB}}}$	0.42	0.33	0.50	1.00	7320
$\sigma_{p_{\text{IN}}^{\text{HRV}}}$	0.51	0.25	0.78	1.00	3717
$\sigma_{p_{\text{IN}}^{\text{AdV}}}$	0.24	0.14	0.34	1.00	5458
$\sigma_{p_{\text{IN}}^{\text{CoV}}}$	0.50	0.45	0.54	1.00	8201
$\sigma_{p_{\text{IN}}^{\text{MPV}}}$	0.40	0.27	0.53	1.00	7352
$\sigma_{p_{\text{IN}}^{\text{PIV}}}$	0.30	0.15	0.45	1.00	5224

ESS is the effective sample size, i. e. the number of independent MCMC samples with estimation power equivalent to the total number of autocorrelated samples[14], and $\hat{R}$ is the Gelman-Rubin convergence diagnostic[15]. Low ESS or $\hat{R}$ or $\hat{R} > 1.10$ indicate bad convergence of the model[16].

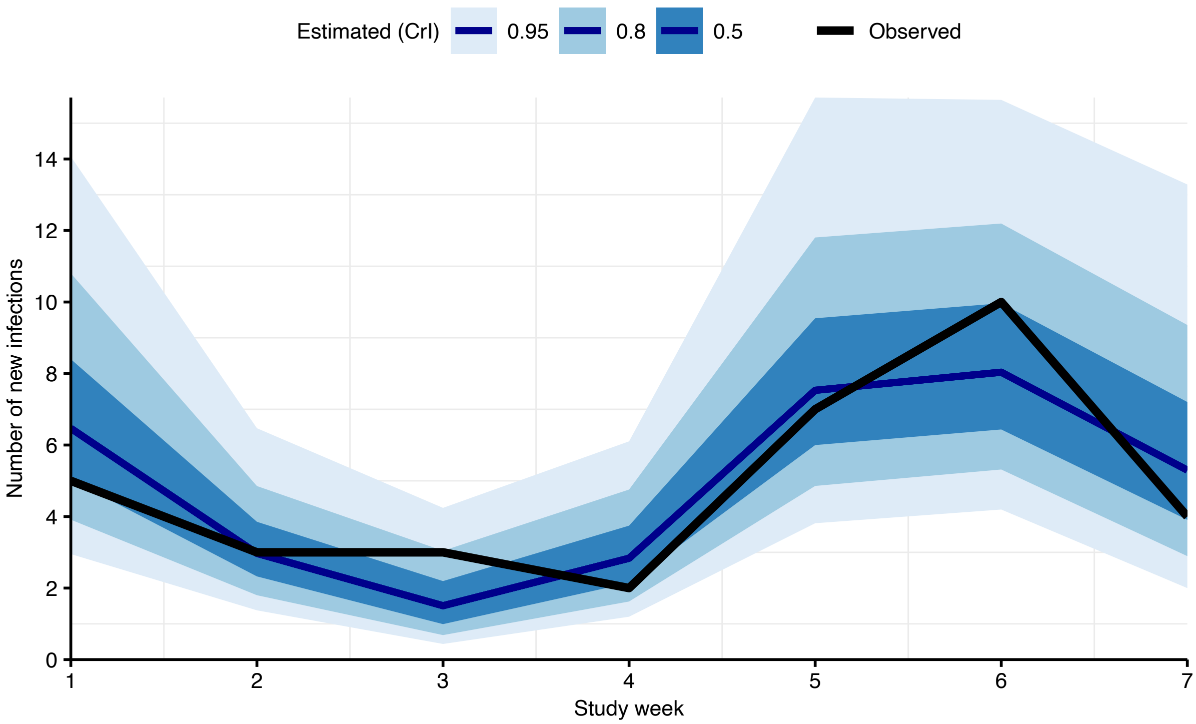

Figure S5. Model-estimated number of new respiratory cases (posterior mean as red line and 50%-, 80%, and 95%-CrIs as shaded areas) and the observed number of new respiratory cases (black line) across classes by study week.

J Detailed results for cough frequency

Differences in the frequency of coughs between classes suggest an association with class-specific virus patterns. Figure S6 shows the relative risk of cough for each respiratory virus. Cough appeared more frequent for IFB and less frequent for AdV.

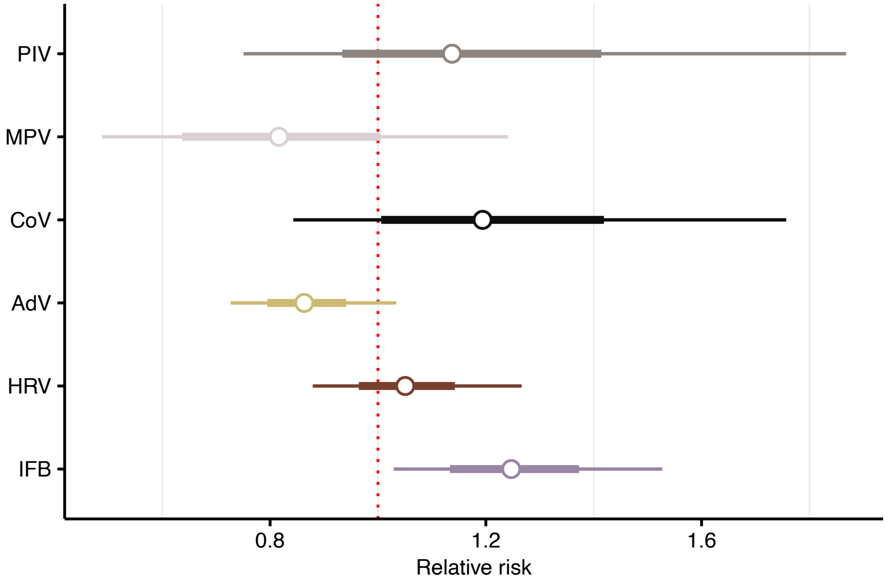

Figure S6. Estimated association (relative risk; posterior mean as dot, 50%, 80%, and 95%-CrI as lines) between cough frequency and number of positive saliva test results by respiratory virus. IFB: Influenza B, HRV: rhinovirus, AdV: adenovirus, CoV: SARS-CoV-2, MPV: metapneumovirus, PIV: parainfluenza virus.

K Possible transmission networks

The distribution of positive saliva samples varied between classes. For example, all but one sample was positive for adenvovirus in class A during the first three study weeks, while the vast majority of positive influenza B samples were found in class B. To illustrate possible transmission chains within classes, we linked positive saliva samples of the same virus that were less than one week apart. Based on this spatiotemporal analysis, we identified 10 possible transmission chains (Figure S7). The longest potential transmission chains occurred in January, referring to a cluster of adenovirus infections in class A and influenza B infections in class B. Molecular genotyping to verify the proposed transmission network was unsuccessful because we could not amplify and sequence any of the gene targets. We also analyzed 105 bioaerosol samples and detected in two of them viral RNA (1 rhinovirus in class A and 1 adenovirus in class B). Similarly, we detected 1 influenza B, 1 rhinovirus, 1 adenovirus, and 1 SARS-CoV-2 in the 20 swabs taken from each filter of an air cleaner after each intervention phase (160 swabs in total).

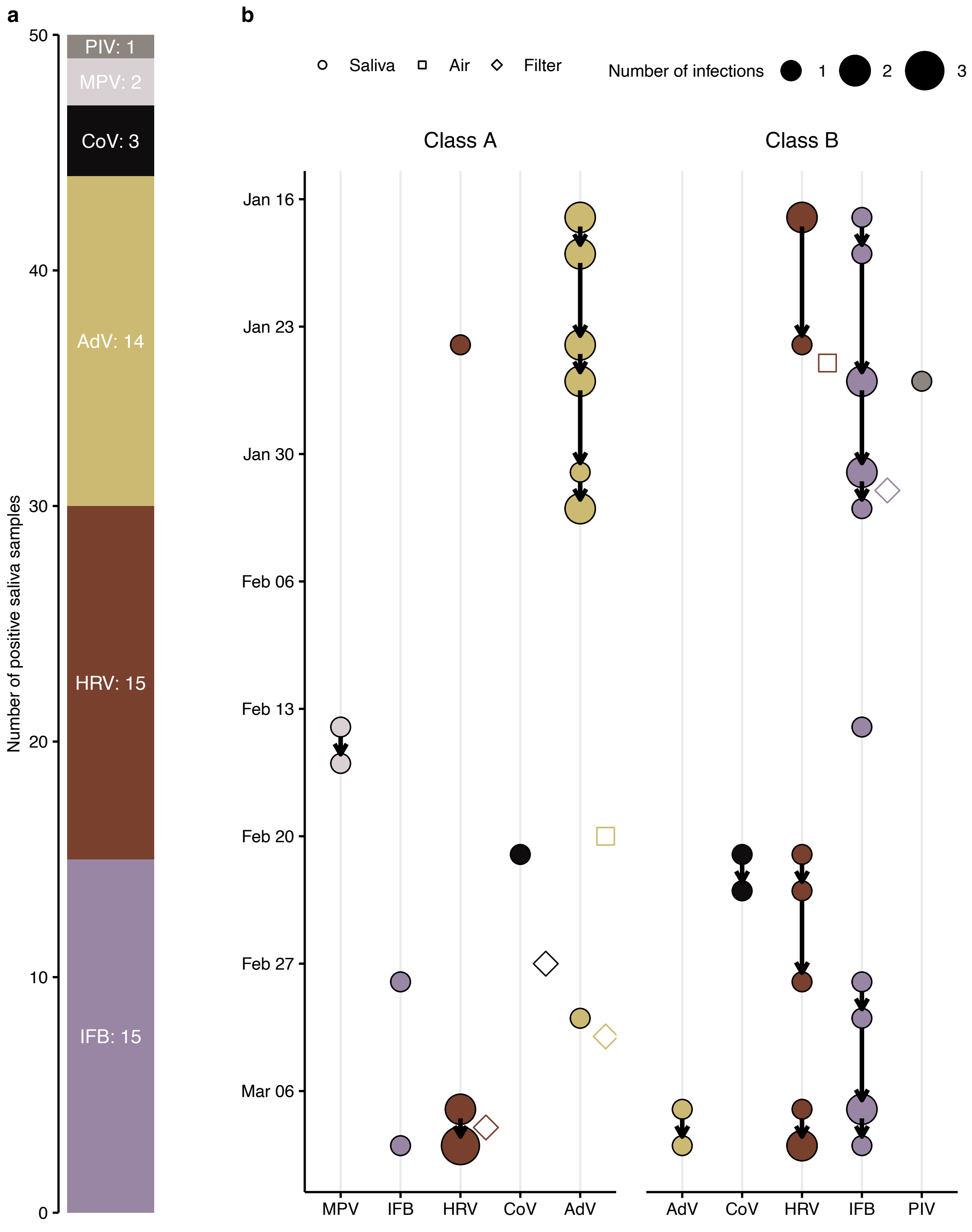

Figure S7. Molecular detection of respiratory viruses and transmission network based on spatiotemporal analysis of students' saliva samples. **(a)** Number of positive saliva samples by virus. **(b)** Daily number of positive saliva samples (colored circles) and possible transmission chains within classes (directed arrows). Positive samples are linked if they belong to the same virus and are less than 1 week apart. Positive samples from the air and filters as blank squares aligned. IFB: influenza B, HRV: human rhinovirus, AdV: adenovirus, CoV: SARS-CoV-2, MPV: human metapneumovirus, PIV: parainfluenza virus.

References

1. Nelson PR, Sears SB, Heavner DL. Application of methods for evaluating air cleaner performance. Indoor Environment. 1993;2(2):111–117. doi:10.1177/1420326X9300200207.

2. Küpper M, Asbach C, Schneiderwind U, Finger H, Spiegelhoff D, Schumacher S. Testing of an indoor air cleaner for particulate pollutants under realistic conditions in an office room. Aerosol and Air Quality Research. 2019;19(8):1655–1665. doi:10.4209/aaqr.2019.01.0029.

3. Bertschinger S, Fenner L, Denecke K. Feasibility of cough detection and classification using artificial intelligence in an ambulatory setting with a ceiling mounted microphone. To appear in the Proceedings of the 36th IEEE International Symposium on Computer-Based Medical Systems (CBMS). 2023;June 2023, L'Aquila, Italy.

4. Harris PA, Taylor R, Thielke R, Payne J, Gonzalez N, Conde JG. Research electronic data capture (REDCap) — A metadata-driven methodology and workflow process for providing translational research informatics support. J Biomed Inform. 2009;42(2):377–381. doi:10.1016/j.jbi.2008.08.010.

5. Harris PA, Taylor R, Minor BL, Elliott V, Fernandez M, O'Neal L, et al. The REDCap consortium: Building an international community of software platform partners. J Biomed Inform. 2019;95:103208. doi:10.1016/j.jbi.2019.103208.

6. McAloon C, Collins A, Hunt K, Barber A, Byrne AW, Butler F, et al. Incubation period of COVID-19: A rapid systematic review and meta-analysis of observational research. BMJ Open. 2020;10(8):e039652. doi:10.1136/bmjopen-2020-039652.

7. Lessler J, Reich NG, Brookmeyer R, Perl TM, Nelson KE, Cummings DA. Incubation periods of acute respiratory viral infections: a systematic review. Lancet Infect Dis. 2009;9(5):291–300. doi:10.1016/S1473-3099(09)70069-6.

8. Gelman A. Scaling regression inputs by dividing by two standard deviations. Statistics in Medicine. 2008;27(15):2865–2873. Available from: https://onlinelibrary.wiley.com/doi/10.1002/sim.3107. doi:10.1002/sim.3107.

9. Gelman A, Jakulin A, Pittau MG, Su YS. A weakly informative default prior distribution for logistic and other regression models. The Annals of Applied Statistics. 2008;2(4):1360–1383. doi:10.1214/08-AOAS191.

10. Gelman A, Hill J, Vehtari A. Regression and other stories. Cambridge University Press; 2020.

11. Stan Development Team. Prior choice recommendations; 2020. Available from: https://github.com/stan-dev/stan/wiki/Prior-Choice-Recommendations [last accessed 2023-04-27].

12. Gabry J, Goodrich B. Prior Distributions for rstanarm models; 2023. Available from: https://cran.r-project.org/web/packages/rstanarm/vignettes/priors.html [last accessed 2023-05-15].

13. Batterman S. Review and extension of CO2-based methods to determine ventilation rates with application to school classrooms. International Journal of Environmental Research and Public Health. 2017;14(2):145. doi:10.3390/ijerph14020145.

14. Stan Development Team. Stan modeling language users guide (Version 2.21); 2022. Available from: https://mc-stan.org/docs/2_21/reference-manual/effective-sample-size-section.html [last accessed 2023-02-15].

15. Gelman A, Rubin DB, others. Inference from iterative simulation using multiple sequences. Stat Sci. 1992;7(4):457–472. doi:10.1214/ss/1177011136.

16. Gelman A, Carlin JB, Stern HS, Dunson DB, Vehtari A, Rubin DB. Bayesian data analysis. Chapman & Hall; 2013.
